# RORA Targeting PRNP Modulates Age‐Related Cataract via Activation Oxidative Injury‐Induced Cellular Senescence and Apoptosis of Lens Epithelial Cells

**DOI:** 10.1111/acel.70547

**Published:** 2026-05-20

**Authors:** Yue Zou, Wanqian Li, Jiaojiao Zhang, Chao Cen, Wanqiu Zheng, Ruonan Li, Hong Cheng, Liang Liang, Juan Kang, Wenjuan Wan, Ke Hu, Shijie Zheng

**Affiliations:** ^1^ Department of Ophthalmology The First Affiliated Hospital of Chongqing Medical University Chongqing China; ^2^ Chongqing Key Laboratory of Prevention and Treatment on Major Blinding Diseases Chongqing China; ^3^ Chongqing Eye Institute Chongqing China; ^4^ Chongqing Branch (Municipality Division) of National Clinical Research Center for Ocular Diseases Chongqing China; ^5^ The People's Hospital of Yubei District of Chongqing City Chongqing China; ^6^ Changdu People's Hospital of Xizang Xizang Changdu China

**Keywords:** age‐related cataract, apoptosis, cellular senescence, oxidative injury, PRNP, RORA

## Abstract

Age‐related cataract (ARC) is a severe vision‐impairing disorder primarily caused by oxidative stress‐induced senescence and apoptosis of lens epithelial cells (LECs). In this study, a sodium selenite‐induced oxidative stress cataract model in neonatal rats was established to simulate the pathological progression of ARC. We found that retinoic acid receptor‐related orphan receptor α (RORA) exacerbates cellular senescence and oxidative damage by targeting prion protein (PRNP), and its small‐molecule inhibitor SR3335 exhibits therapeutic potential in regulating ARC progression. In vitro experiments showed that inhibiting RORA significantly alleviated cellular senescence, enhanced the anti‐apoptotic capacity of LECs, and improved their resistance to oxidative stress, whereas activating RORA exerted opposite effects. In vivo, intravitreal injection of recombinant PRNP protein was demonstrated to abrogate the protective effect of RORA silencing, thereby exacerbating the progression of ARC. Mechanistically, RNA sequencing and dual‐luciferase reporter assay revealed that RORA binds to its downstream target PRNP. RORA targets PRNP to regulate the p53/p21/Bax signaling pathway, thereby suppressing both cellular senescence and apoptosis. These findings highlight the critical role of the transcription factor RORA in ARC development by modulating oxidative stress injury, apoptosis, and senescence in LECs. The identification of PRNP as a downstream target of RORA may provide a novel dual‐target strategy for ARC treatment.

## Graphical abstract

The molecular mechanisms of RORA targeting PRNP activate oxidative injury‐induced cellular senescence and apoptosis of lens epithelial cells. Current research confirms that the transcription factor RORA targets PRNP, exacerbating cellular senescence and apoptosis of LECs (Figure 7). The RORA‐PRNP‐p53/p21/Bax axis may offer an innovative framework for identifying potent therapeutic targets for ARC and other age‐related ocular diseases. All these molecular mechanisms were created with BioGDP.com.

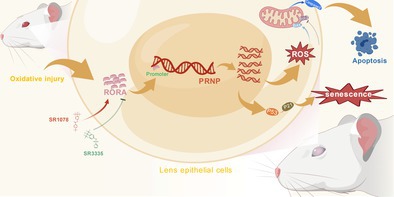

## 1 Introduction

Age‐related cataract (ARC) is one of the most common causes of reversible vision loss worldwide (Asbell et al. 2005; Liu et al. 2017). Currently, the primary clinical treatment for cataracts is surgery (Liu et al. 2024). With the accelerating aging of the global population, preventing the formation of cataractous lens is the way to avoid the occurrence of postoperative complications such as refractive error and posterior capsular opacification (PCO) from the source (Lin et al. 2016; Wei et al. 2024).

Lens epithelial cells (LECs) are monolayers of cells that surround the anterior capsule and equatorial region of the lens. Their normal structure and function are vital for maintaining transparency and the metabolic dynamics of the entire lens in equilibrium (Mathias et al. 2010). During aging, LECs are accompanied by adverse events such as oxidative DNA damage and protein accumulation due to misfolding (Periyasamy and Shinohara 2017). In addition, the massive accumulation of reactive oxygen species (ROS) and significant disturbance of redox homeostasis result in LECs loss and damage, causing the opacification of the lens and ultimately ARC (Periyasamy and Shinohara 2017; Ma et al. 2023). These studies suggest that oxidative stress‐induced cellular senescence and apoptosis in LECs are considered to be a central factor in the development of ARC (Tjahjono et al. 2020; Hayashi et al. 2018; Wei et al. 2021). Therefore, further investigations into the damage caused by oxidative stress in LECs might lead to new approaches for cataract therapy and prevention.

Retinoic acid receptor‐associated orphan receptors (RORs), ligand‐mediated transcription factors of the nuclear receptor superfamily, consist of three members: RORA, RORB, and RORC (Green et al. 2008; Kojetin and Burris 2014). RORs regulate numerous biological processes, such as autophagy, mitophagy, oxidative stress, and inflammation (Nematisouldaragh et al. 2024). In the eye, RORs have been identified as new targets for retinal vascular and ocular degenerative diseases due to their regulation of normal lens and retinal development (Yemanyi et al. 2023), as well as their aberrant expression affecting the development of a variety of ocular diseases such as oxygen‐induced retinopathy (OIR) (Sun et al. 2017) and age‐related macular degeneration (AMD) (Akula et al. 2024). Retinoic acid receptor‐related orphan nuclear receptor α (RORA) is widely expressed in ocular tissues such as lens, retinal ganglion cells, bipolar cells, and photoreceptors (Ladurner et al. 2021), whereas its activation of γF‐crystallin gene expression to stimulate the differentiation of LECs into fibroblasts has drawn our particular attention (Tini et al. 1995). Currently, numerous studies have focused on the role of RORA in regulating oxidative stress in age‐degenerative diseases: in heart failure, RORA reduces oxidative stress by regulating the melatonin‐activated antioxidant enzyme MnSOD to exert an antihypertrophic effect (Xu et al. 2019); in Parkinson's disease, RORA protects neurons from oxidative stress by reducing mitochondrial ROS production (Al‐Zaid et al. 2023); while in human clinical studies, RORA has been linked to a higher risk of wet (neovascular) age‐related macular degeneration (Schaumberg et al. 2010).

Despite its established role in regulating oxidative stress‐induced injury in a variety of age‐related diseases, the role of RORA in ARC remains unclear. In the present study, we observed specific upregulation of RORA in anterior capsule membrane tissues of cataract patients, H_2_O_2_‐induced oxidative damage cell model and Na_2_SeO_3_‐induced cataract rat model. In vitro, the effects of the knockdown and activation of RORA on oxidative stress, senescence and apoptotic phenotype of LECs were confirmed; in vivo, RORA inhibition rescued lens opacification in cataract rats. The RNA‐seq and dual‐luciferase reporter assay indicated that the prion protein gene (PRNP) is a downstream target of RORA. The protective effect of RORA inhibition was reversed in both in vitro and in vivo by PRNP overexpression.

## 2 Materials and Methods

### 2.1 Study Participants

Applying the following criteria, we collected anterior capsular membrane tissue from 8 individuals with age‐related cataracts: (1) 45–60 years of age; (2) nuclear cataract ≥ N3 or cortical cataract ≥ C3 (CNP cataract categorization); and (3) no prior history of systemic diseases or ocular disorders. Five age‐matched normal controls were also enrolled. The Ethics Committee of the First Affiliated Hospital of Chongqing Medical University approved this study (No. 2023‐01), and we affirm that it was conducted in strict compliance with the Declaration of Helsinki's principles. The informed consent form was obtained from each participant. The tissue samples were stored in liquid nitrogen for subsequent experiments immediately after surgical isolation.

### 2.2 Animal Model and Treatment

Healthy 8‐day‐old Sprague–Dawley suckling rats were acquired from Chongqing Medical University's Experimental Animal Center and housed in a standard‐compliant experimental environment. Neonatal SD rat pups were used randomly regardless of sex, without sex matching or separation. Following the protocol for the cataract model successfully established by our team previously (Gao et al. 2025), rat pups received sodium selenite (Na_2_SeO_3_, 20 μmol/kg, Sigma‐Aldrich, USA) subcutaneously in the nape of the neck on postnatal days 10, 12, and 14 to establish a cataract model. From postnatal day 10 to day 16, the RORA small‐molecule chemical inhibitor SR3335 (15 mg·kg^−1^·day^−1^ i.p. for 7 days; TargetMol, USA) was intraperitoneally injected 1 h prior to the Na_2_SeO_3_ injection (Rajput et al. 2017). The control group was injected with an equivalent volume of vehicle in the same manner. On postnatal day 21, cataract images of the rats were obtained via ultrasound‐guided slit‐lamp biomicroscopy under anesthesia with intraperitoneal injection of 1.25% Tribromoethanol (TBE). The anterior segment of the eye was imaged using optical coherence tomography of the anterior segment of the eye (OCT; VG200D, SVision Imaging Ltd., Henan, China), and all the animals were subsequently euthanized. Ocular tissue samples were dissected and collected. The animal experiment was approved by the Ethics Committee of the First Affiliated Hospital of Chongqing Medical University (No. 2024‐0835). All personnel involved in the experiment received training. The ARVO Statement on the Use of Animals in Ophthalmic and Vision Research was adhered to throughout all experimental procedures.

### 2.3 Intravitreal Injection

PRNP Protein, Rat, Recombinant (His) as a recombinant PRNP protein (50 μg·kg^−1^; TargetMol, USA) was intravitreally injected into anesthetized rats on postnatal day 12 at a volume of 1 μL per eye, while the control group was injected with the same volume of vehicle solution. Topical antibiotic ointment was applied to the ocular surface for three consecutive days after the injection.

### 2.4 Hematoxylin and Eosin (H&E) Staining

The collected intact eyeball tissues were first fixed in 4% paraformaldehyde, followed by paraffin embedding, and then stained into 4–6 μm sections with hematoxylin–eosin (H&E) as directed by the manufacturer. Light microscopy (Leica, Germany) was then used to observe histological changes of the ocular tissues.

### 2.5 Cell Culture and Treatment

The SRA01/04 human lens epithelial cell line was obtained from Procell Life Science & Technology Co. Ltd. (Wuhan, China). Dulbecco's modified Eagle medium (DMEM, Gibco, USA) containing 10% fetal bovine serum (FBS, Gibco, USA) supplemented with 1% penicillin–streptomycin solution (Gibco, USA) was used. The cells were incubated at 37°C in a humidified 5% CO_2_ incubator. To generate an oxidative stress microenvironment, the cells were treated with 200 μM H_2_O_2_ for 24 h. To verify cellular senescence, after 24 h of stimulation, the culture medium was replaced with fresh complete medium and cultured for another 24 h to perform a short recovery period after stress removal. SR1078 (10 μM; TargetMol, USA) was administered half an hour prior to H_2_O_2_ treatment.

### 2.6 Cell Transfection

Three small interfering RNAs (siRNAs) targeting RORA were obtained from Tsingke Biotechnology Co. Ltd. When the cells reached 70% confluency, the transfection reagent Lipofectamine 2000 (Invitrogen, USA) and si‐RORA (50 nM) were mixed and diluted in serum‐free DMEM to form transfection complexes, which were subsequently added to the cells. After 4–6 h, the entire medium was replaced, and the transfection efficiency was evaluated by observing the transfected cells under a fluorescence microscope. Cell samples were harvested for subsequent experiments between 18 and 48 h later.

### 2.7 Western Blot (WB)

RIPA lysis buffer (Beyotime, China) with a 1% protease inhibitor cocktail (Beyotime, China) was used to generate protein lysates for protein sample extraction. The BCA Protein Assay Kit (Beyotime, China) was used to calculate the protein concentrations of the collected samples. After separation by 4%–20% SDS‐PAGE, the proteins were transferred to PVDF membranes (Millipore, USA) via a semidry transfer system (Bio‐Rad, USA) and blocked with 5% non‐fat milk for 2 h at room temperature, followed by incubation with primary antibodies against Bax (1:5000, Proteintech), Bcl‐2 (1:1000, Proteintech), Cleaved‐Caspase3 (1:1000, Cell Signaling Technology), P53 (1:1000, Abmart), P21 (1:1000, Abmart), Lamin B1 (1:1000, Abmart), P16 (1:1000, Abmart), His tag (1:1000, Huabio), RORA (1:1000, Proteintech) and PRNP (1:1000, Proteintech). The membranes were incubated at 4°C for 16–18 h. After washing three times with TBST for 10 min each time, the membranes were incubated with HRP‐affinipure goat anti‐rabbit IgG (1:8000, Proteintech) or HRP‐affinipure goat anti‐mouse IgG (1:8000, Proteintech) for 1 h at room temperature, and washed again with TBST. Relative protein expression levels were normalized to the internal control β‐actin (1:5000, Proteintech). The immunoblot bands were detected with an enhanced chemiluminescence (ECL) kit (MedChemExpress, USA) and quantified with ImageJ software.

### 2.8 Reverse Transcription–Quantitative PCR (RT–qPCR)

TRIzol (Invitrogen, USA) was used to extract total RNA, and the RT Master Mix for Quantitative PCR (qPCR) Kit (MedChemExpress, USA) was used to reverse‐transcribe the extracted RNA into complementary DNA (cDNA). RT‐qPCR was performed with SYBR Green qPCR Master Mix (MedChemExpress, USA) on the 7500 Fast Real‐Time PCR system (ABI, USA). The 2^−ΔΔCt^ method was used to calculate the relative expression levels of target genes after normalization to β‐actin levels. The primers were synthesized by Shanghai Sangon Co. Ltd., and are shown in Table S1.

### 2.9 Detection of Malondialdehyde (MDA)

The malondialdehyde (MDA) content was measured by the thiobarbituric acid (TBA) colorimetric assay, utilizing an MDA detection kit (Nanjing Jiancheng Bioengineering Institute, China) in strict compliance with the manufacturer's guidelines. The brief procedure was as follows: the collected cells or tissues were homogenized by ultrasonic disruption on ice to get homogeneous extracts; afterwards, the prepared samples or standard solutions were added into the corresponding working solution and incubated at 95°C for at least 1 h. After being cooled to room temperature and centrifuged, the absorbance of the supernatant was detected at a wavelength of 532 nm with a microplate reader. The MDA concentration of each sample was calculated based on the standard curve, and the final results were normalized to the total protein content of the corresponding sample.

### 2.10 Detection of Superoxide Dismutase (SOD)

The activity of superoxide dismutase (SOD) was quantitatively determined by the water‐soluble tetrazolium salt (WST‐8) colorimetric method. With the SOD activity detection kit (Beyotime, China), tissue homogenates or cell lysates were added to the working solution and reaction initiation solution in sequence according to the kit instructions. The mixture was then placed in a constant temperature incubator and incubated at 37°C for 30 min. After incubation, the absorbance was measured at 450 nm. One unit (U) of SOD activity was defined as the amount of enzyme needed to inhibit 50% of the WST‐8 reduction reaction. The total SOD activity was normalized to the protein concentration of the sample, and the results were expressed as U/mg protein.

### 2.11 JC‐1 Mitochondrial Membrane Potential Assay

The JC‐1 kit (Beyotime, China) was used to detect the mitochondrial membrane potential in step with the manufacturer's instructions. Each group of cells was incubated with JC‐1 staining solution for 20 min at 37°C, then washed three times with 1 × JC‐1 Staining Buffer and immediately observed and imaged under a fluorescence microscope (Leica, Germany).

### 2.12 Detection of Reactive Oxygen Species (ROS)

To perform intracellular ROS experiments, the SRA01/04 cells were incubated with a 10 μM DCFH‐DA ROS working probe at 37°C for 15 min. After sufficient washing with phosphate‐buffered saline (PBS) three times for 5 min each, the intracellular ROS levels were measured with an inverted fluorescence microscope (Leica, Germany). Fluorescence intensity was quantified using ImageJ software to determine intracellular ROS levels.

### 2.13 Cell Viability Assay

Cell viability was evaluated via the Cell Counting Kit‐8 (CCK‐8) assay (Dojindo, Japan). After adding 10 μL of CCK‐8 solution to each well, the plates were incubated at 37°C for 2 h. A microplate reader (Thermo Fisher Scientific, USA) was used to measure the absorbance at 450 nm.

### 2.14 Annexin V‐FITC/Propidium Iodide (PI) Assay

Quantitative assessment of cell apoptosis was conducted using an Annexin V‐FITC apoptosis detection kit (Beyotime, China). In brief, cells exposed to 200 μM H_2_O_2_ were collected and resuspended in 1 × Annexin V binding buffer. Next, 5 μL of Annexin V‐FITC and 10 μL of PI staining solution were added to the cell suspension, which was then incubated in the dark at room temperature for 15 min. After staining was completed, the cells were analyzed with a Thermo Fisher Scientific Attune NxT flow cytometer to assess the apoptosis rate.

### 2.15 Dual‐Luciferase Reporter Assay

Deletion mutations were constructed for the top three predicted potential binding sites in the PRNP promoter, which were identified using the JASPAR database. Cells were co‐transfected with the PRNP‐luc reporter plasmid, RORA overexpression plasmid, Renilla luciferase reporter plasmid, and empty vector plasmid, and then cultured for 48 h. Luciferase activities were detected using a Dual‐Luciferase Reporter Assay Kit (Beyotime, China) in accordance with the manufacturer's instructions. Briefly, the cell samples were collected after adding lysis buffer; subsequently, Firefly Luciferase Assay Reagent was added to measure the relative light units (RLU). Afterwards, Renilla Luciferase Assay Working Solution was added to the same samples to determine the RLU again. The final luciferase activity was calculated as the ratio of RLU measured by Firefly Luciferase to that measured by Renilla Luciferase.

### 2.16 Senescence‐Associated β‐Galactosidase (SA‐β‐Gal) Staining

Senescent cells and tissues were detected using the Senescence β‐Galactosidase Staining Kit (Beyotime, China) according to the manufacturer's instructions. Briefly, cultured cells and frozen sections of the eyeball were washed once with phosphate‐buffered saline (PBS) and fixed with 1× Fixation Buffer for 10 min at room temperature. After fixation, samples were rinsed twice with PBS and subsequently incubated with the β‐Galactosidase Staining Working Solution at 37°C in a dry incubator (without CO_2_ supplementation) for approximately 48 h. Following staining, cellular samples were imaged on a Leica Mateo TL imaging system to acquire bright‐field micrographs, and tissues were imaged under a fluorescence microscope (Leica, Germany).

### 2.17 5‐Ethynyl‐2′‐Deoxyuridine (Edu) Assay

Cells were seeded at 2 × 10^4^ cells per well in a 24‐well plate containing a pre‐placed coverslip. After a 2‐h incubation with 2× Edu working solution (Beyotime, China), the cells were fixed, permeabilized, and incubated with the Click‐iT reaction mixture for 30 min at room temperature in the dark. Subsequently, the nuclei were counterstained with Hoechst, and the stained cells were imaged via fluorescence microscopy (Leica, Germany).

### 2.18 RNA Sequencing (RNA‐Seq)

High‐quality RNA samples collected from the siRORA + H_2_O_2_ group and the vehicle + H_2_O_2_ group were rigorously controlled via an Agilent 2100 bioanalyzer followed by library construction. Differential gene expression was analyzed according to standard Illumina sequence analysis protocols. The above process was supervised and performed by Novo Biotechnology Co. Ltd. (Beijing). The identification of differentially expressed genes (DEGs) was based on *p*adj < 0.05 and |log2(fold change) (log2FC)| > 1.

### 2.19 Statistical Analysis

Statistical analysis was performed via SPSS Statistics 26.0 (IBM, USA). All experiments included at least three biological replicates. Student's *t*‐test was used for comparisons between two groups, and one‐way ANOVA was used for comparisons of three or more groups. Nonparametric tests were used for rating the severity of cataracts under the slit lamp: the Mann–Whitney *U* test for two samples and the Kruskal–Wallis *H* test for multiple samples. *p* value < 0.05 was considered statistically significant.

## 3 Results

### 3.1 RORA Is Upregulated in the Na_2_SeO_3_ ‐Induced Cataract Rat Model

**FIGURE 1:**
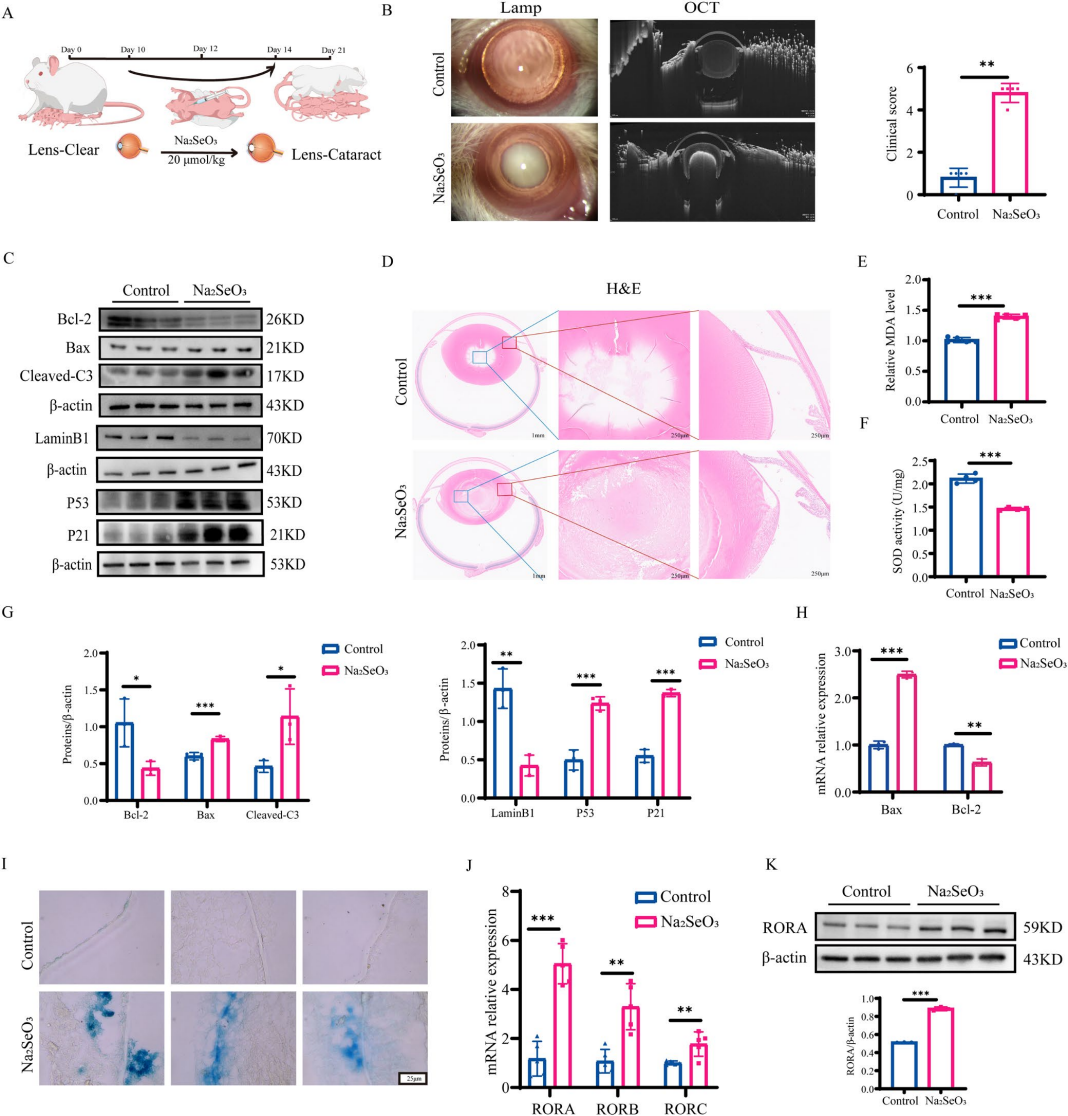
RORA is upregulated in the Na_2_SeO_3_‐induced ARC rat model. (A) Schematic diagram of the in vivo experimental design. (B) Slit‐lamp microscopy images and OCT images of cataract rat lens from the control and Na_2_SeO_3_ groups. Clinical scoring based on slit‐lamp examination results (*n* = 5). **, *p* < 0.01; Mann–Whitney *U* test. (C, G) Quantification of relative protein expression of Bcl‐2, Bax, Cleaved‐C3, Lamin B1, P21, and P53 (*n* = 3). *, *p* < 0.05; **, *p* < 0.01; ***, *p* < 0.001; unpaired Student's *t*‐test. (D) H&E‐stained images of rat eyeballs from the control and Na_2_SeO_3_ groups (*n* = 5). Scale bars: 1 mm (left panels), 250 μm (middle and right panels). (E, F) MDA content and SOD activity in lens tissues from the control and Na_2_SeO_3_ groups (*n* = 4–5). ***, *p* < 0.001; unpaired Student's *t*‐test. (H) Relative mRNA levels of apoptosis markers (Bax, Bcl‐2) in lens tissues measured by RT‐qPCR (*n* = 3). **, *p* < 0.01; ***, *p* < 0.001; unpaired Student's *t*‐test. (I) SA‐β‐gal staining of the control group and sodium selenite group (*n* = 3). Scale bar: 25 μm. (J) Relative mRNA expression of ROR family members in lens tissues from the control and Na_2_SeO_3_ groups (*n* = 5). **, *p* < 0.01; ***, *p* < 0.001; unpaired Student's *t*‐test. (K) Quantification of relative RORA protein expression (*n* = 3). ***, *p* < 0.001; unpaired Student's *t*‐test.

We constructed an ARC rat model by subcutaneous injection of Na_2_SeO_3_ (20 μmol/kg) into the dorsal neck on postnatal days 10, 12, and 14 (Figure 1A) and scored cataract severity according to the grading system established by Hiraoka and Clark (1995). Optical coherence tomography (OCT) and slit lamp biomicroscopy (slit lamp) were used to evaluate lens characteristics in live rats, with corresponding clinical scores assigned (Figure 1B). Slit lamp examination showed that, compared with the control group, the Na_2_SeO_3_‐treated group exhibited nuclear damage and lens opacification in rats. OCT examination revealed that Na_2_SeO_3_ produced a large, dense region inside the lens that impeded light transmission. Pathological sections of the eyeball revealed severe pathological alterations, including vacuolation in the equatorial area and the disrupted structure in the nuclear region of the lens (Figure 1D). All indicators reflect that sodium selenite disrupts the clear and transparent physiological state of the lens and causes severe aging changes—cataracts. At the molecular level, we first measured malondialdehyde (MDA) levels and superoxide dismutase (SOD) activity and the results showed that Na_2_SeO_3_ stimulation significantly increased oxidative injury in the LECs of cataract rats (Figure 1E,F). Then, we evaluated the expression levels of aging molecules (Lamin B1, P53, and P21) and apoptosis markers (Bcl‐2, Bax, and Cleaved‐Caspase3) indicating that oxidative stress induced cellular senescence and apoptosis (Figure 1C,G,H). Meanwhile, we performed SA‐β‐gal staining on frozen sections of rat eyeballs. The acellular transparent basement membrane shown in the figure is the lens capsule, to which lens epithelial cells are attached (Figure 1I). Significantly increased SA‐β‐gal positivity evidenced that, compared with the control group, lens from sodium selenite–induced cataract rats exhibited marked cellular senescence. These results confirm severe senescence and apoptosis in lens tissue in the sodium selenite–induced SD rat cataract model. Consistently, we observed that the RNA expression levels of the ROR family in LECs were upregulated (Figure 1J). We further measured the protein expression level of RORA—the most significantly upregulated member of the ROR family—and observed that it was elevated as previously expected (Figure 1K). These findings suggest that RORA was increased during lens aging and apoptosis.

### 3.2 RORA Is Upregulated in ARC Lens Capsules and H_2_O_2_ ‐Induced LECs

**FIGURE 2:**
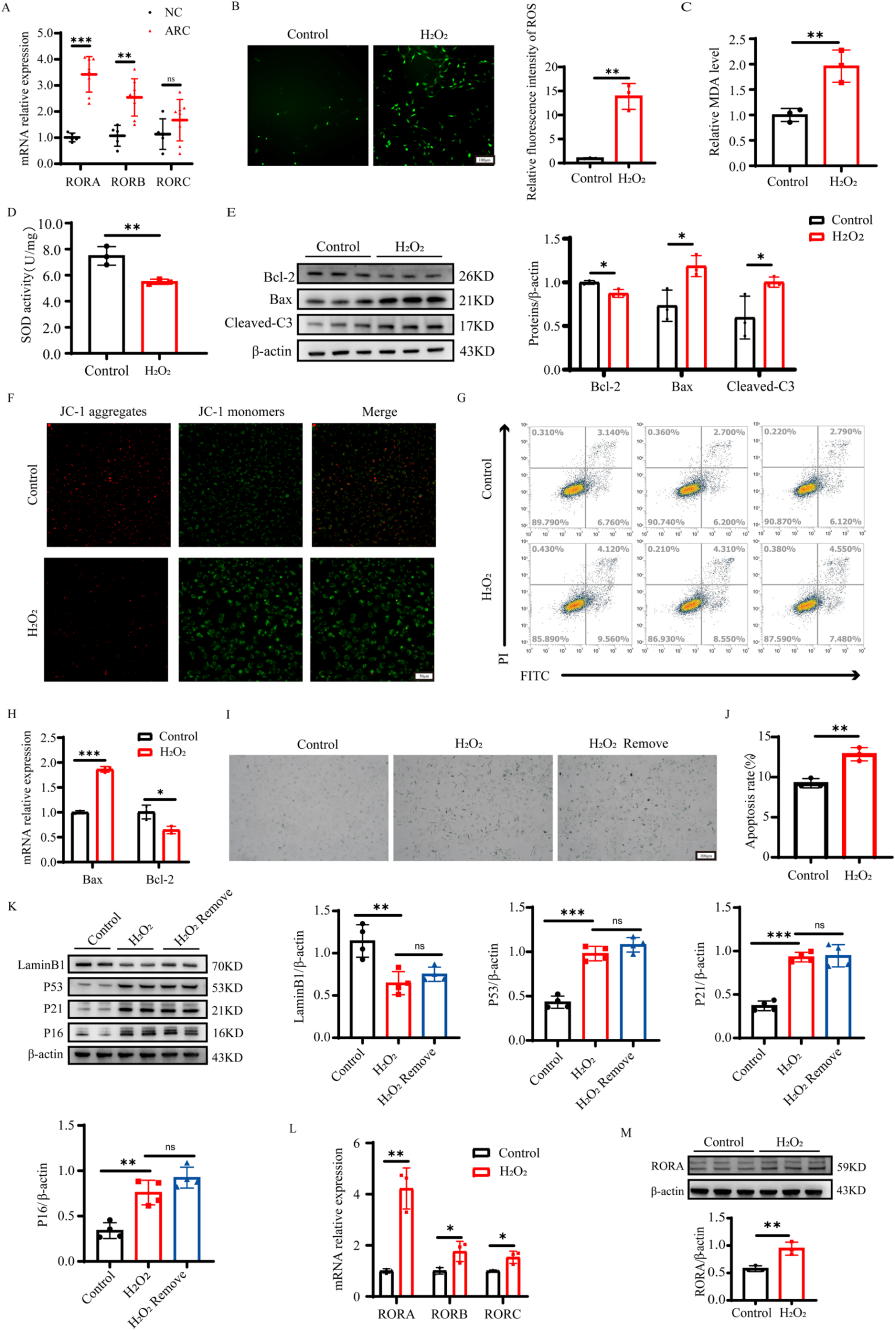
RORA is upregulated in ARC lens capsules and H_2_O_2_‐induced LECs. (A) Relative mRNA expression of ROR family members (RORA, RORB, RORC) in anterior lens capsule tissues from normal controls (*n* = 5) and ARC patients (*n* = 8), detected by RT‐qPCR. **, *p* < 0.01; ***, *p* < 0.001; ns, *p* > 0.05; unpaired Student's *t*‐test. (B) ROS levels in LECs with or without stimulation by 200 μM H_2_O_2_ (*n* = 3). **, *p* < 0.01; unpaired Student's *t*‐test. Scale bar, 100 μm. (C, D) MDA content and SOD activity in LECs from the control and H_2_O_2_ groups (*n* = 3). **, *p* < 0.01; unpaired Student's *t*‐test. (E) Protein expression levels of Bcl‐2, Bax, and Cleaved‐C3 in LECs treated with or without H_2_O_2_, detected by Western blot (*n* = 3). *, *p* < 0.05; unpaired Student's *t*‐test. (F) Mitochondrial membrane potential in the control and H_2_O_2_ groups detected by JC‐1 staining (*n* = 3). Scale bar, 50 μm. (G, J) Apoptosis rates were determined by flow cytometry in the control and H_2_O_2_ groups (*n* = 3). **, *p* < 0.01; unpaired Student's *t*‐test. (H) Relative mRNA levels of Bax and Bcl‐2 in LECs measured by RT‐qPCR (*n* = 3). *, *p* < 0.05; ***, *p* < 0.001; unpaired Student's *t*‐test. (I) SA‐β‐gal staining of each group (*n* = 3). Scale bar, 100 μm. (K) Protein expression levels of Lamin B1, P53, P21, and P16 in LECs treated with or without H_2_O_2_, detected by Western blot (*n* = 4). ns, *p* > 0.05; **, *p* < 0.01; ***, *p* < 0.001; one‐way ANOVA. (L) Relative mRNA expression of ROR family members in the control and H_2_O_2_ groups analyzed by RT‐qPCR (*n* = 3). *, *p* < 0.05; **, *p* < 0.01; unpaired Student's *t*‐test. (M) Quantification of relative protein expression of RORA in LECs treated with or without H_2_O_2_ (*n* = 3). **, *p* < 0.01; unpaired Student's *t*‐test.

First, we performed testing on human samples. Compared with those in normal controls, the RNA expression levels of the ROR family genes in the lens capsule tissues of cataract patients generally tended to increase, with RORA being the most significant (Figure 2A). To investigate the function and mechanism of RORA in LECs, we established an in vitro LEC oxidative damage model using 200 μM H_2_O_2_ for 24 h. First, ROS production was observed under an inverted fluorescence microscope, and the results showed that H_2_O_2_ stimulation significantly increased oxidative injury in LECs (Figure 2B). The increase in the MDA content and the decrease in SOD activity also revealed that H_2_O_2_ induced oxidative damage to LECs (Figure 2C,D). Furthermore, a decrease in the mitochondrial membrane potential was observed with the JC‐1 probe (Figure 2F), which indicates an increase in early apoptosis. Flow cytometric analysis showed a significant increase in the total number of apoptotic cells in the H_2_O_2_‐treated group compared with the control group (Figure 2G,J). Moreover, Western blot and RT‐qPCR analysis revealed that the expression of the antiapoptotic molecule Bcl‐2 was down‐regulated and that the expression of the pro‐apoptotic molecules Bax and Cleaved‐Caspase3 was upregulated (Figure 2E,H). Besides, compared with the control group, the number of SA‐β‐gal‐positive cells was markedly increased after H_2_O_2_ treatment and remained elevated 24 h after H_2_O_2_ withdrawal, indicating persistence of the senescence‐associated phenotype during the short recovery period (Figure 2I). Accordingly, we observed sustained upregulation of P53, P21, and p16INK4a as well as persistent loss of Lamin B1 in both the H_2_O_2_‐stimulated group and the short‐term recovery group after stimulus removal (Figure 2K). Furthermore, we performed Edu cell proliferation assays, which demonstrated that H_2_O_2_ stimulation induced cell cycle arrest in LECs (Figure S2G). Collectively, oxidative stress injury induced by H_2_O_2_ triggers cellular senescence and exacerbates the apoptotic process in LECs. Additionally, we observed the variations in ROR family RNA expression (Figure 2L) and confirmed the increase in RORA protein expression (Figure 2M), which was consistent with the upregulation trend observed in clinical cataract lens capsule samples and in vivo experiments. These findings suggest that oxidative stress, senescence and apoptosis in LECs are closely associated with the abnormally high expression of RORA.

### 3.3 Knockdown and Activation of RORA Alter H_2_O_2_ ‐Induced Cellular Senescence, Apoptosis and Oxidative Damage In Vitro

**FIGURE 3:**
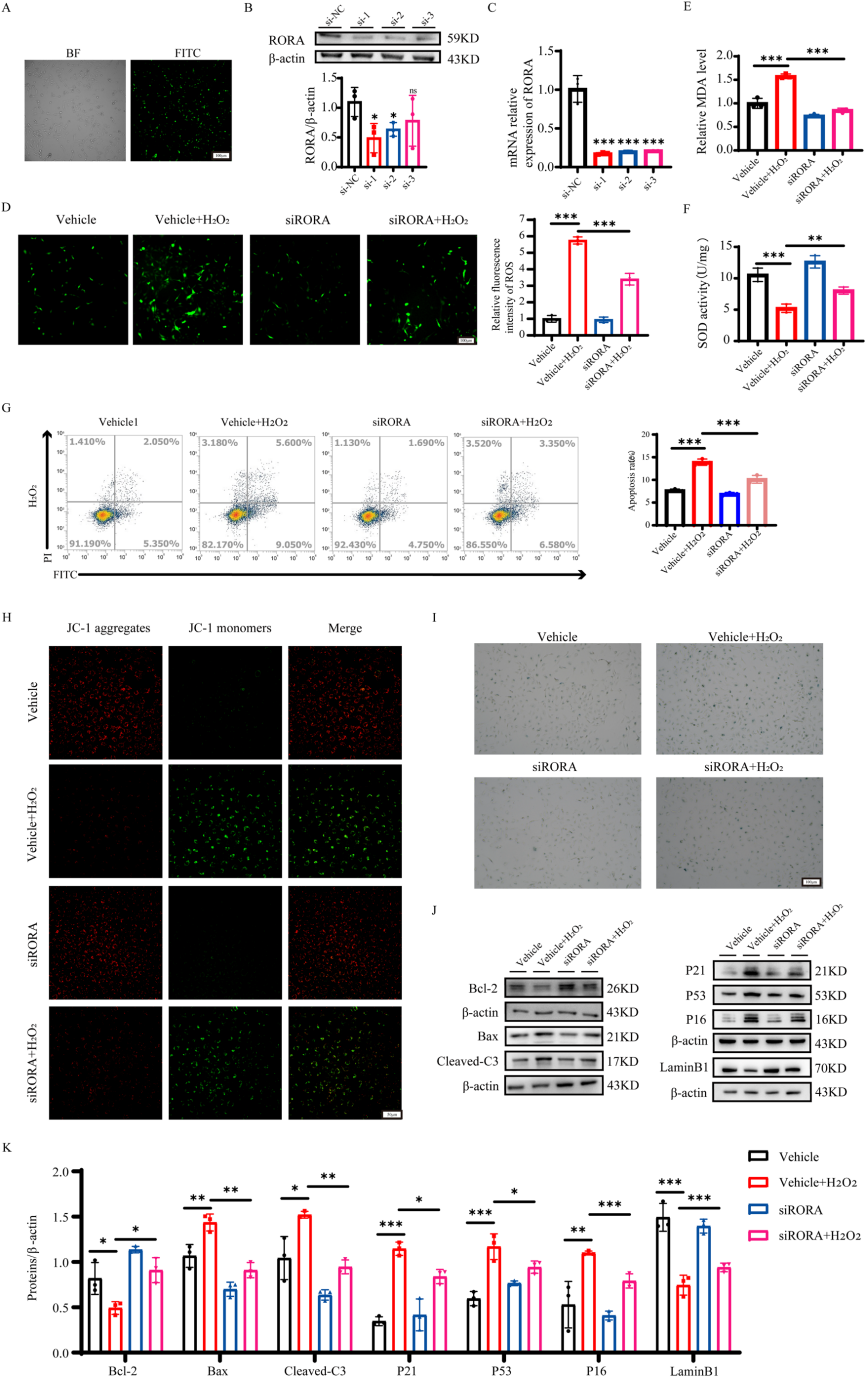
RORA knockdown attenuates H_2_O_2_‐induced cellular senescence, apoptosis and oxidative damage in vitro. (A) Fluorescent expression in SRA01/04 cells transfected with lentivirus and white light control. Scale bar, 100 μm. (B, C) Transfection efficiency assessed by Western blot and RT‐qPCR (*n* = 3). ns, *p* > 0.05; *, *p* < 0.05; ***, *p* < 0.001; unpaired Student's *t*‐test. (D) Fluorescence images and relative fluorescence intensity analysis of ROS levels in the four groups: Vehicle, vehicle + H_2_O_2_, siRORA, and siRORA + H_2_O_2_ groups (*n* = 3). Scale bar, 100 μm. ***, *p* < 0.001; one‐way ANOVA. (E, F) MDA and SOD levels in the four groups (*n* = 3). **, *p* < 0.01; ***, *p* < 0.001; one‐way ANOVA. (G) Apoptosis rates were determined by flow cytometry in each group (*n* = 3). ***, *p* < 0.001; one‐way ANOVA. (H) Mitochondrial membrane potential measured by JC‐1 in each of the four groups (*n* = 3). Scale bar, 50 μm. (I) SA‐β‐gal staining of each group (*n* = 3). Scale bar, 100 μm. (J) Relative protein expression levels in the four groups (*n* = 3). *, *p* < 0.05; **, *p* < 0.01; ***, *p* < 0.001; one‐way ANOVA.

Based on the finding that RORA is elevated in response to oxidative stress both in vitro and in vivo, we investigated the role of RORA via dual intervention treatment in the H_2_O_2_‐induced LECs model. First, we constructed a siRORA plasmid and transfected it at a concentration of 50 nM to knock down RORA, and the resulting images confirmed the optimal cell status and infection effectiveness (Figure 3A). In addition, via WB and RT‐qPCR experiments, we screened the siRORA‐1 plasmid with a superior knockdown efficiency for further research (Figure 3B,C). Second, we used the SR1078 to activate RORA. The CCK‐8 assay was used to evaluate the cytotoxic effects of SR1078. The results revealed that 10 μM SR1078 is close to the IC_50_ value (Figure S1A). RT‐qPCR and Western blot analysis confirmed the activation of RORA by SR1078 at the protein and RNA levels (Figure S1B,C). Compared with H_2_O_2_ treatment alone, ROS experiments verified that RORA knockdown reduced the level of oxidative stress in LECs (Figure 3D), whereas RORA activation further increased the degree of oxidative stress (Figure S1D). MDA content levels (Figure 3E and Figure S1F) and SOD activity (Figure 3F and Figure S1G) further supported these findings. Furthermore, inhibition of RORA expression significantly alleviated stress‐induced apoptosis (Figure 3G). We followed up with mitochondrial membrane potential experiments, and the findings once more showed a variation in consistency (Figure 3H,E and Figure S1E). Besides, RORA knockdown decreased the percentage of SA‐β‐gal‐positive cells (Figure 3I) and alleviated cell cycle arrest (Figure S2H). Additionally, the expression levels of senescence and apoptosis proteins were regulated after RORA knockdown and activation (Figure 3J,K and Figure S1H), demonstrating that RORA impacts the senescence and apoptosis of LECs caused by oxidative damage. Collectively, these findings suggest that RORA may act as a key regulator of the functional phenotype of LECs by aggravating cellular senescence, reducing LECs' defense against apoptosis, and lowering their resistance to oxidative stress.

### 3.4 PRNP Is a Downstream Target of RORA

**FIGURE 4:**
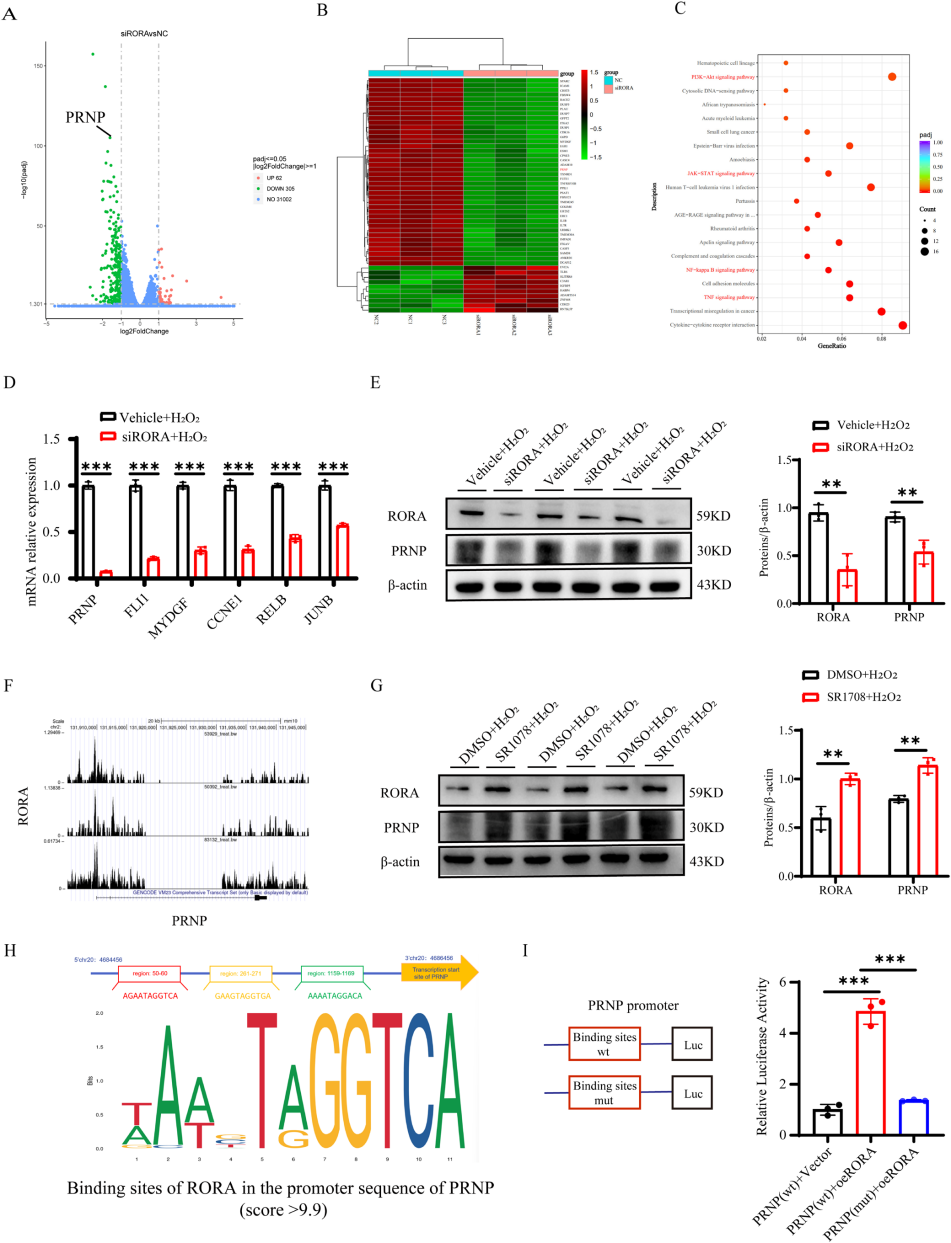
PRNP is a downstream target of RORA. (A) Volcano plot of differentially expressed genes (DEGs) between the vehicle + H_2_O_2_ and siRORA + H_2_O_2_ groups (|log_2_FC| ≥ 1, *p*adj ≤ 0.05). (B) Heatmap showing the expression profiles of the 50 most significantly dysregulated DEGs between the vehicle + H_2_O_2_ and siRORA + H_2_O_2_ groups (*n* = 3). (C) KEGG pathway enrichment analysis of DEGs in the vehicle + H_2_O_2_ and siRORA + H_2_O_2_ groups. (D) RT‐qPCR validation of the mRNA expression of six candidate target genes (including PRNP) to confirm RNA‐seq results (*n* = 3). ***, *p* < 0.001; unpaired Student's *t*‐test. (E, G) RORA and PRNP protein expression levels in each group, verified by Western blot (*n* = 3). **, *p* < 0.01; unpaired Student's *t*‐test. (F) ChIP‐seq genomic tracks showing RORA binding peaks in the promoter region of PRNP (data from Cistrome Data Browser). (H) Schematic of potential RORA binding sites in the PRNP promoter, predicted by JASPAR database (score > 9.9). (I) The relative luciferase activity from wild‐type and mutant‐type‐transfected cells was measured by a dual luciferase reporter system (*n* = 3). ***, *p* < 0.001; one‐way ANOVA.

To investigate the molecular mechanism by which RORA knockdown modulates the LEC phenotype, we performed RNA sequencing (RNA‐seq) on cells with or without the transfection of siRORA before H_2_O_2_ stimulation. The volcano plot revealed 367 differentially expressed genes (DEGs), 305 of which were downregulated and 62 of which were upregulated (|log2FC| ≥ 1, *p*adj ≤ 0.05) (Figure 4A). The cluster heatmap depicts the cluster analysis between the 50 most significantly dysregulated genes (Figure 4B). Furthermore, the Kyoto Encyclopedia of Genes and Genomes (KEGG) enrichment revealed that RORA knockdown markedly enriched several aging‐ and apoptosis‐related pathways, such as the PI3K‐Akt, JAK–STAT, NF‐κB, and TNF signaling pathways (Figure 4C). On the basis of senescence and apoptosis, we focused on screening six candidate target genes and verified them via RT‐qPCR; the expression of the PRNP gene was the most dramatically downregulated (Figure 4D). Then, we first measured PRNP mRNA expression in anterior capsular samples from cataract patients. The results showed that PRNP was significantly upregulated, consistent with the expression pattern of RORA (Figure S2A). Subsequently, increased PRNP mRNA and protein levels were also observed in H_2_O_2_‐induced cell models and sodium selenite‐induced animal models (Figure S2B–E). These results suggest that PRNP may be a downstream target of RORA. Furthermore, to verify the correlation between PRNP expression and RORA activity, we examined PRNP protein expression after RORA knockdown and found that PRNP was significantly downregulated (Figure 4E), whereas PRNP expression was upregulated following RORA activation (Figure 4G). Moreover, RORA, as a transcription factor, may regulate the transcriptional efficiency of genes by binding to specific DNA sequences in the promoter region. To investigate whether RORA directly targets PRNP, we first analyzed data from the Cistrome Data Browser (http://cistrome.org/db/#/), which revealed significant binding peaks of RORA in the PRNP promoter region (Figure 4F). Further analysis using the JASPAR database (https://jaspar.elixir.no/) identified three high‐confidence candidate binding sites (score > 9.9) between RORA and the PRNP promoter, based on their conserved sequence motifs (Figure 4H). We further performed a dual‐luciferase reporter assay. The results showed that the PRNP promoter containing the binding sites (PRNP‐wt) was significantly activated by RORA expression, whereas this activation was markedly attenuated when the binding sites were deleted by mutation (PRNP‐mut) (Figure 4I). This experiment provides direct evidence that the transcription factor RORA binds to the PRNP promoter region and regulates its transcription, which suggests that PRNP is a downstream target of RORA, and that RORA may influence the pathogenesis of ARCs by directly regulating PRNP transcription.

### 3.5 Overexpression of PRNP Reverses RORA Knockdown‐Induced Phenotypic Alterations in LECs In Vitro

**FIGURE 5:**
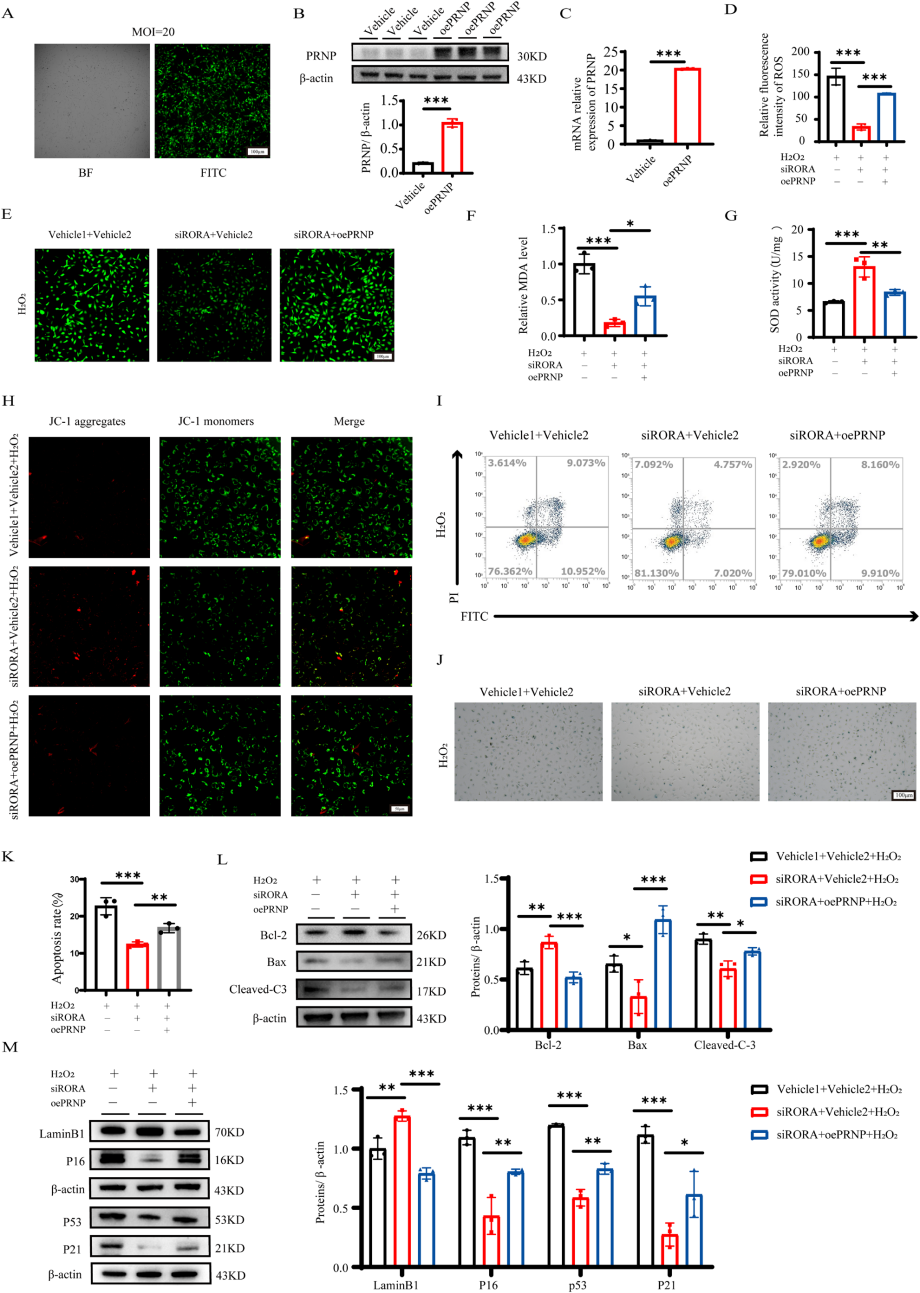
Overexpression of PRNP reverses RORA knockdown‐induced phenotypic alterations in LECs in vitro. (A) White light and fluorescence images following lentivirus transfection. Scale bar, 100 μm. (B, C) The knockdown efficiency of PRNP was evaluated via Western blot and RT‐qPCR (*n* = 3). ***, *p* < 0.001; unpaired Student's *t*‐test. (D, E) Fluorescence images and relative fluorescence intensity analysis of ROS levels in the three groups: Vehicle1 + Vehicle2 + H_2_O_2_ group, siRORA + Vehicle2 + H_2_O_2_ group, and siRORA + oePRNP + H_2_O_2_ group (*n* = 3). Scale bar, 100 μm. ***, *p* < 0.001; one‐way ANOVA. (F, G) MDA content and SOD activity in the three groups (*n* = 3). *, *p* < 0.05; **, *p* < 0.01; ***, *p* < 0.001; one‐way ANOVA. (H) Mitochondrial membrane potential in the three groups detected by JC‐1 staining (*n* = 3). Scale bar, 50 μm. (I, K) Apoptosis rates were determined by flow cytometry in each group (*n* = 3). **, *p* < 0.01; ***, *p* < 0.001; one‐way ANOVA. (J) SA‐β‐gal staining of each group (*n* = 3). Scale bar, 100 μm. (L, M) Protein expression levels in the three groups, detected by Western blot (*n* = 3). *, *p* < 0.05; **, *p* < 0.01; ***, *p* < 0.001; one‐way ANOVA.

To clarify whether PRNP is involved in RORA‐mediated cellular senescence and apoptosis, we applied a lentivirus to overexpress PRNP. Transfection was set up at an MOI = 20 after we first screened the treatment conditions with lentivirus (Figure 5A). We confirmed the strong effectiveness of overexpressing PRNP via WB and RT‐qPCR studies (Figure 5B,C). We subsequently separated the SRA01/04 cells into three groups on the basis of the various intervention methods: the vehicle1 + vehicle2 + H_2_O_2_ group, the siRORA + vehicle2 + H_2_O_2_ group, and the siRORA + oePRNP + H_2_O_2_ group. In the H_2_O_2_‐induced cellular environment, ROS tests demonstrated that the overexpression of PRNP markedly reversed the decrease in the level of cellular oxidative stress levels caused by RORA inhibition (Figure 5D,E). Moreover, both the MDA and SOD experiments confirmed that the overexpression of PRNP inhibited the enhanced antioxidant capacity of SRA01/04 after H_2_O_2_ stimulation (Figure 5F,G). Additionally, the overexpression of PRNP in the RORA‐silenced LECs decreased their capacity to resist apoptosis and aging, as evidenced by a decrease in the mitochondrial membrane potential (Figure 5H), an increase in the total apoptotic level of cells (Figure 5I,K), an increase in the percentage of SA‐β‐gal‐positive cells (Figure 5J), cell cycle arrest (Figure S2I), downregulation of Bcl‐2 and Lamin B1, and upregulation of Bax, Cleaved‐Caspase3, P16, P21, and P53 (Figure 5L,M). These results indicate that the protective effects of RORA silencing are reversed by upregulating PRNP expression.

### 3.6 Recombinant PRNP Protein Reverses the Protective Effect of RORA Inhibition in Na_2_SeO_3_ ‐Induced Cataract Rats In Vivo

**FIGURE 6:**
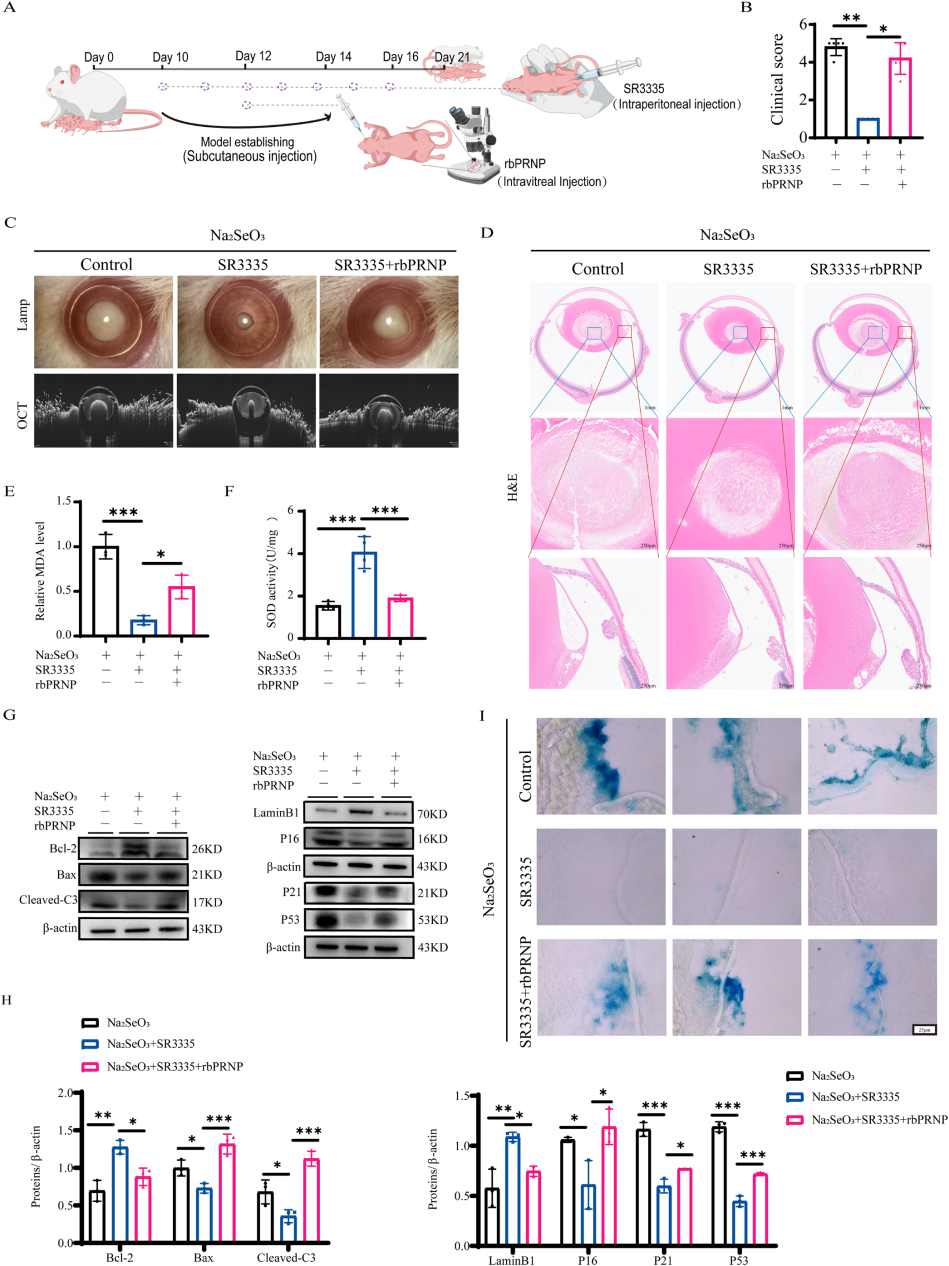
Recombinant PRNP protein reverses the protective effect of RORA inhibition in Na_2_SeO_3_‐induced cataract rats in vivo. (A) Schematic diagram of the in vivo experimental design. (B, C) Slit‐lamp images, OCT images, and statistical chart of cataract grading in rat lens from the Na_2_SeO_3_, Na_2_SeO_3_ + SR3335, and Na_2_SeO_3_ + SR3335 + rbPRNP (recombinant PRNP) groups (*n* = 5). *, *p* < 0.05; **, *p* < 0.01; Kruskal–Wallias *H* test. (D) H&E‐stained sections of rat eyeballs from the three groups (*n* = 5). Scale bars: 1 mm (top panels), 250 μm (middle and bottom panels). (E, F) MDA content and SOD activity in lens tissues from the three groups (*n* = 3–4). *, *p* < 0.05; ***, *p* < 0.001; one‐way ANOVA. (G, H) Protein expression levels in the three groups, detected by Western blot (*n* = 3). *, *p* < 0.05; **, *p* < 0.01; ***, *p* < 0.001; one‐way ANOVA. (I) SA‐β‐gal staining of each group (*n* = 3). Scale bar, 25 μm.

To validate the regulatory role of RORA in cataract pathogenesis, we administered the RORA‐specific inhibitor SR3335 via intraperitoneal injection in a Na_2_SeO_3_‐induced cataract rat model. To confirm that PRNP functions as a downstream effector of RORA, we increased the expression of PRNP by intravitreal injection of the recombinant PRNP protein in vivo (Figure 6A). The rats were divided into three groups: the Na_2_SeO_3_ group, the Na_2_SeO_3_ + SR3335 group, and the Na_2_SeO_3_ + SR3335 + rbPRNP group. Once the eyes of all the rats had opened, we performed slit lamp examination, OCT imaging on the rats, and separated the eyeballs for histological analysis with H&E staining. Following rbPRNP treatment, rats treated with Na_2_SeO_3_ + SR3335 had significantly worsened lens opacity and light scattering (Figure 6B,C). Similarly, the pathological structure clearly differed (Figure 6D). PRNP supplementation worsened the oxidative stress phenotype of LECs, according to the MDA and SOD data (Figure 6E,F). In addition, the upregulation of PRNP expression reversed the change in the protein expression levels of lens apoptosis (Figure 6G,H). Furthermore, we observed that protein expression of p16, P21, and P53 was significantly decreased after RORA inhibition and significantly rescued by PRNP recombinant protein treatment, whereas Lamin B1 expression showed the opposite pattern (Figure 6G,H). Similarly, SA‐β‐gal positivity was significantly reduced under RORA inhibition and markedly restored upon PRNP administration (Figure 6I). Collectively, these findings demonstrate that PRNP can reverse the protective effect generated by RORA silencing, confirming that PRNP is a critical downstream mediator of RORA in ARC pathogenesis.

### 3.7 The Molecular Mechanisms of RORA Targeting PRNP Activates Oxidative Injury‐Induced Cellular Senescence and Apoptosis of Lens Epithelial Cells

**FIGURE 7:**
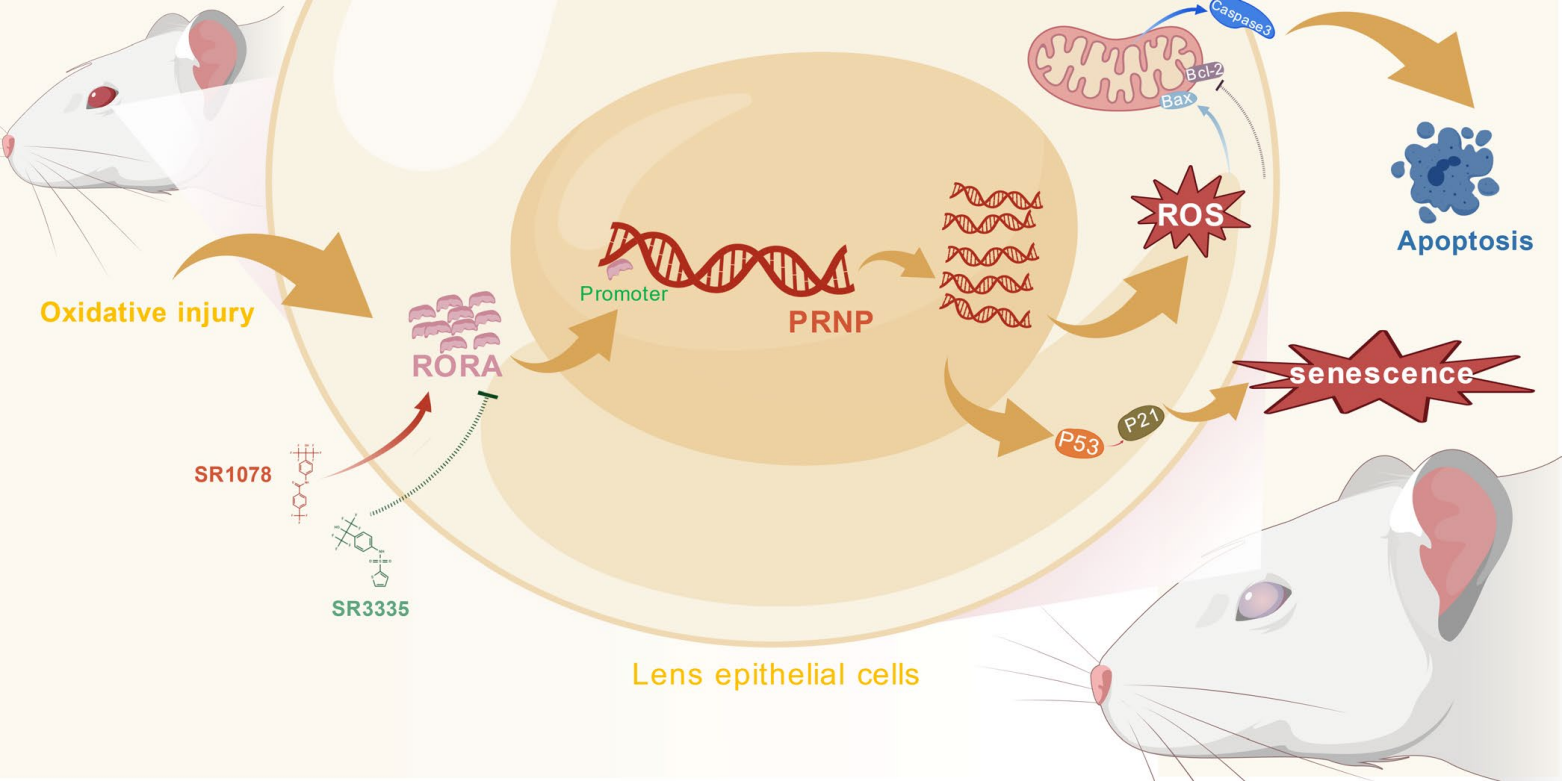
The molecular mechanisms of RORA targeting PRNP activate cellular senescence and apoptosis of lens epithelial cells.

Current research confirms that the transcription factor RORA targets PRNP, exacerbating cellular senescence and apoptosis of LECs (Figure 7). The RORA‐PRNP‐p53/p21/Bax axis may offer an innovative framework for identifying potent therapeutic targets for ARC and other age‐related ocular diseases. All these molecular mechanisms were created with BioGDP.com (Jiang et al. 2025).

## 4 Discussion

In this study, we demonstrated that the transcription factor RORA is significantly upregulated in lens epithelial cells (LECs) under oxidative stress. This finding was consistently observed across three experimental settings: anterior capsule tissues isolated from cataract patients, a Na_2_SeO_3_‐induced cataract rat model, and an H_2_O_2_‐stimulated SRA01/04 cell model. To dissect RORA's functional role, we performed dual‐intervention experiments (knockdown/activation) in vitro. Results showed that RORA knockdown alleviated H_2_O_2_‐induced cellular senescence and apoptosis in LECs, whereas RORA activation exacerbated these pathological processes. In vivo, pharmacological inhibition of RORA (using SR3335) in the Na_2_SeO_3_‐induced cataract rat model significantly reduced lens opacity and improved histological features of the lens. Mechanistically, we employed whole‐transcriptome sequencing to identify downstream mediators of RORA. This screen prioritized the prion protein gene (PRNP) as a candidate target. Dual‐luciferase reporter assays confirmed that the PRNP promoter could be specifically bound and transcriptionally activated by RORA. Subsequent validation confirmed that PRNP overexpression in vitro reversed the protective effects of RORA knockdown. In vivo, administration of recombinant PRNP protein aggravated lens opacity and pathological damage in cataract model rats—further supporting PRNP's role as a downstream effector of RORA. Collectively, these data establish RORA as a key regulator of cellular senescence and apoptosis in LECs and highlight its potential as a therapeutic target for cataract.

Age‐related cataracts (ARCs) and other age‐associated ocular diseases are emerging as major public health challenges (Asbell et al. 2005). Maintenance of LEC homeostasis is critical for preventing ARC progression. Previous studies have identified two core drivers of ARC: excessive reactive oxygen species (ROS) production in LECs, which induces aging and triggers apoptosis, and impairment of the cellular antioxidant defense system (Pendergrass et al. 2005). To investigate these mechanisms, we established complementary in vitro and in vivo oxidative stress models of LEC injury. We assessed oxidative stress via ROS detection, measured malondialdehyde (MDA, a marker of lipid peroxidation) and superoxide dismutase (SOD, a key antioxidant enzyme) levels, and evaluated mitochondrial membrane potential using JC‐1 staining. Consistent with prior reports (Bayir and Kagan 2008), our findings demonstrated that oxidative damage in LECs initiates a cascade of senescence and apoptosis, which is further supported by dysregulated expression of genes linked to this process, such as P53, P21, Bax, Bcl‐2, and Cleaved‐Caspase3. Notably, the sodium selenite–induced neonatal rat cataract model is an acute, oxidative stress–driven model, and its experimental context (neonatal animals) and acute modeling approach do not fully recapitulate the pathological progression of human age‐related cataract (ARC). Notwithstanding this limitation, the sodium selenite model remains a well‐recognized, classic, and widely used tool in cataract research. It reliably replicates key pathogenic hallmarks associated with human senile nuclear cataract, the most prevalent subtype of ARC, including glutathione depletion, cellular senescence, and increased LECs apoptosis (Avarachan and Rawal 1987; Gao et al. 2025; Anon, n.d.). These conserved pathological features validate its utility for investigating oxidative stress–mediated cataract formation, a core pathogenic mechanism of age‐related cataract. In addition, we agree that validation in an aging or adult cataract model would further strengthen the translational relevance of this work and will be an important direction for our future studies.

Retinoic acid receptor‐related orphan receptors (RORs) are a subfamily of the nuclear receptor (NR) superfamily, functioning as ligand‐dependent transcription factors. RORs have been implicated in multiple ocular pathologies, serving as novel targets for retinal vascular development and the treatment of degenerative eye diseases (Yemanyi et al. 2023). We agree that RORA has often been reported to exert cytoprotective effects in several aging‐related contexts, particularly through the regulation of antioxidant defenses and mitochondrial function. In the present study, however, we found that RORA exacerbated oxidative stress and cellular senescence in lens epithelial cells. Although this finding appears contradictory to conventional understanding, it actually reflects the multiple regulatory properties of RORA as a nuclear receptor, including high cell specificity, context dependence, dose dependence, and mechanism specificity of target pathways. Notably, this study is the first to report aberrant ROR expression (particularly RORA upregulation) across ARC patient tissues, in vitro LEC models, and in vivo cataract models. Functional validation in SRA01/04 cells showed that RORA exacerbates oxidative injury and apoptosis in LECs under stress. In vivo, RORA inhibition rescued Na_2_SeO_3_‐induced lens opacity and histological abnormalities. These phenotypic changes strongly support a causal association between RORA and ARC pathogenesis. Although our findings suggest that inhibiting RORA has potential value in delaying lens aging and preventing or mitigating cataract progression, efficient and targeted delivery of RORA inhibitors to lens tissue remains the core bottleneck limiting clinical translation of this strategy. Future studies will focus on the development of lens‐targeted drug delivery systems (Kim et al. 2023; Xu et al. 2018; Zhao et al. 2023), including optimizing delivery technologies such as nanocarriers and ophthalmic sustained‐release formulations to improve drug accumulation and bioavailability in the lens; exploring topical administration and penetration‐enhancing strategies compatible with the ocular physiological environment to reduce systemic exposure and potential side effects; and further integrating biodegradable materials and smart controlled‐release systems to construct safe and long‐acting lens‐targeted delivery platforms. These efforts will provide key technical support for advancing RORA inhibitor–based interventions toward clinical translation.

Prion protein (PrP, encoded by the PRNP gene) is a highly conserved cell surface glycoprotein (Korth et al. 1997; Chrobak et al. 2024) expressed in most vertebrate tissues throughout development (Chrobak et al. 2024). PRNP regulates diverse cellular processes, including oxidative stress responses, senescence and apoptosis (Anon 2007). Previous studies reported PRNP upregulation in oxidative stress contexts—for example, in rat brains following neural probe implantation (Ereifej et al. 2018) and in diabetic cataracts or posterior capsular opacification. Moreover, during skeletal muscle aging, PRNP disrupts muscle homeostasis, induces endoplasmic reticulum stress and age‐related mitochondrial autophagy, and activates mitochondrial oxidative stress and cellular senescence (Liu et al. 2025). Native PRNP is localized to the cell membrane via GPI anchoring, which depends on phosphatidylinositol modification. In this study, the recombinant PRNP protein employed in vivo lacks the necessary components for membrane anchoring and GPI modification, thus losing GPI dependent membrane binding and existing as a soluble free protein. After intravitreal injection, recombinant PRNP forms a high concentration gradient in the avascular gel matrix of the vitreous body, diffuses freely to the anterior lens capsule, and continuously interacts with the membrane of lens epithelial cells (LECs), driving cellular uptake through endocytosis. We have confirmed that recombinant PRNP can enter LECs by detecting the His tag carried by the recombinant protein (Figure S2F). Meanwhile, the vitreous cavity is immune privileged, and PRNP, as an endogenous protein, exhibits low immunogenicity. Therefore, intravitreal injection of recombinant PRNP is generally considered to have good intraocular biocompatibility. Our study, for the first time, by administering PRNP recombinant protein via intravitreal injection, linked the pro‐aging and pro‐stress effects of PRNP to the transcription factor RORA and validated this mechanism using both in vivo and in vitro experiments.

Overall, our research offers new perspectives on how the transcription factor RORA modulates the senescence and apoptosis response of LECs driven by oxidative stress. Future research will explore PRNP's role in RORA‐knockout ARC models and evaluate the therapeutic potential of small‐molecule RORA inhibitors (targeting the RORA‐PRNP axis) for ARC prevention. To sum up, these results may provide a novel approach for determining effective treatment targets for ARC and other age‐related ocular diseases.

## Author Contributions

Y.Z., W.L., K.H., and S.Z. designed the experiments. Y.Z. and W.L. performed the experiments. Y.Z. analyzed the data and wrote the manuscript. Y.Z., S.H., W.W., and K.H. revised the manuscript. J.Z., C.C., and W.Z. helped with the experimental implementation, including the processing of the statistical data. R.L., H.C., L.L., and J.K. helped with clinical sample collection and analysis. S.Z. supervised the research. K.H., W.W., and L.L. acquired funding. All the authors have read and approved the final manuscript.

## Funding

The present study was supported by the National Natural Science Foundation of China (grant nos. 82371098), the Natural Science Foundation of Tibet Autonomous Region [grant nos. XZZR202402100(W)], and the Chongqing Medical Scientific Research Project (Joint project of Chongqing Health Commission and Science and Technology Bureau) (grant nos. 2024ZDXM033 and 2024GDRC005).

## Ethics Statement

All animal experimental protocols were approved by the Animal Ethics Committee of Chongqing Medical University (IACUC‐CQMU‐2024‐0835) and followed the principles of the Declaration of Helsinki. In addition, the study adhered to the Association for Research in Vision and Ophthalmology (ARVO) Statement on the Use of Animals in Ophthalmic and Vision Research.

## Conflicts of Interest

The authors declare no conflicts of interest.

## Supporting information


**Figure S1:** RORA activation exacerbates H_2_O_2_‐induced cellular senescence, apoptosis and oxidative damage in vitro. (A) Viability of LECs treated with increasing concentrations of the RORA activator SR1078, measured by CCK‐8 assay (*n* = 3). *, *p* < 0.05; ***, *p* < 0.001; ns, *p* > 0.05; unpaired Student's *t*‐test. (B, C) RORA activation efficiency assessed by RT‐qPCR (mRNA level) and Western blot (protein level) (*n* = 3). *, *p* < 0.05; **, *p* < 0.01; ***, *p* < 0.001; one‐way ANOVA. (D) Fluorescence images and relative fluorescence intensity analysis of ROS assay results (*n* = 3). Scale bar, 100 μm. ***, *p* < 0.001; one‐way ANOVA. (E) Mitochondrial membrane potential in each of the three groups determined by the JC‐1 probe (*n* = 3). Scale bar, 50 μm. (F, G) MDA content and SOD activity in the three groups: Control, H_2_O_2_ and H_2_O_2_ + SR1078 groups (*n* = 3). *, *p* < 0.05; **, *p* < 0.01; ***, *p* < 0.001; one‐way ANOVA. (H) Relative protein expression levels of apoptosis‐related factors (Bcl‐2, Bax, Cleaved‐C3) and senescence factors (P21, P53) in the three groups (*n* = 3). *, *p* < 0.05; **, *p* < 0.01; ***, *p* < 0.001; one‐way ANOVA.

**Figure S2:** (A) Relative mRNA expression of PRNP in anterior lens capsule tissues from normal controls (*n* = 5) and ARC patients (*n* = 8), detected by RT‐qPCR. ***, *p* < 0.001; unpaired Student's *t*‐test. (B) Relative mRNA expression of PRNP in Control and H_2_O_2_ groups (*n* = 4). **, *p* < 0.01; unpaired Student's *t*‐test. (C) Relative mRNA expression of PRNP in Control and Na_2_SeO_3_ groups (*n* = 5). ***, *p* < 0.001; unpaired Student's *t*‐test. (D, E) Relative protein expression of PRNP in each group (*n* = 3). **, *p* < 0.01; unpaired Student's *t*‐test. (F) Relative protein expression of His‐PRNP in rats with or without rbPRNP (*n* = 3). ***, *p* < 0.001; unpaired Student's *t*‐test. (G, H, I) The Edu assays were used to analyze the proliferation of cells in each group (*n* = 3). Scale bar, 100 μm.

**Table S1:** RT‐qPCR primers used in this study.

## Data Availability

All data needed to evaluate the conclusions in the paper are presented in the paper and/or the Supporting Information. The RNA‐seq data has been deposited in the Sequence Read Archive (SRA) under the accession number PRJNA1467257.
