# Supplementary figures and images for: RORA Targeting PRNP Modulates Age‐Related Cataract via Activation Oxidative Injury‐Induced Cellular Senescence and Apoptosis of Lens Epithelial Cells
Source: Aging Cell. 2026 May 20;25(6):e70547. doi: 10.1111/acel.70547 (PMC13239784; doi:10.1111/acel.70547)

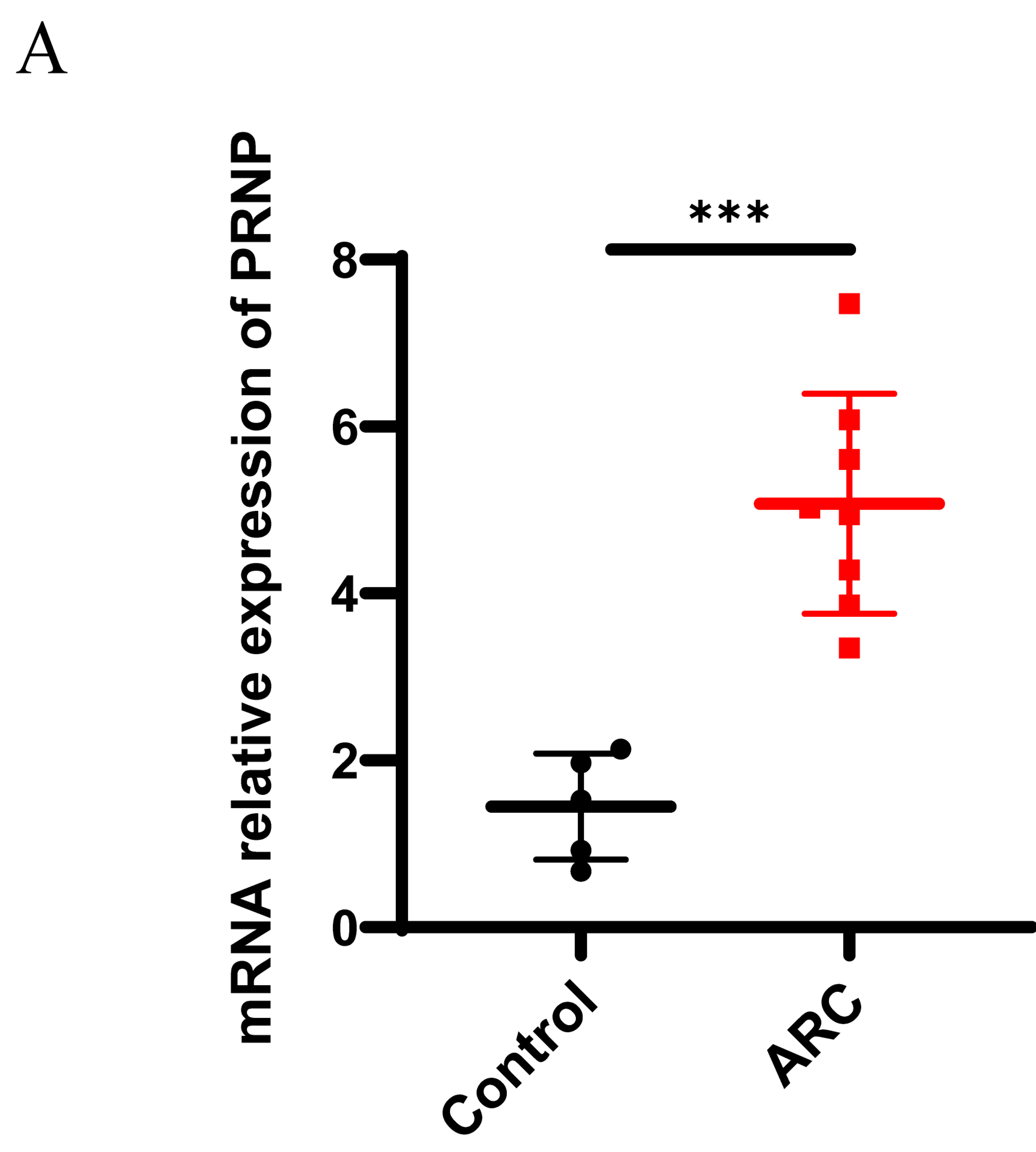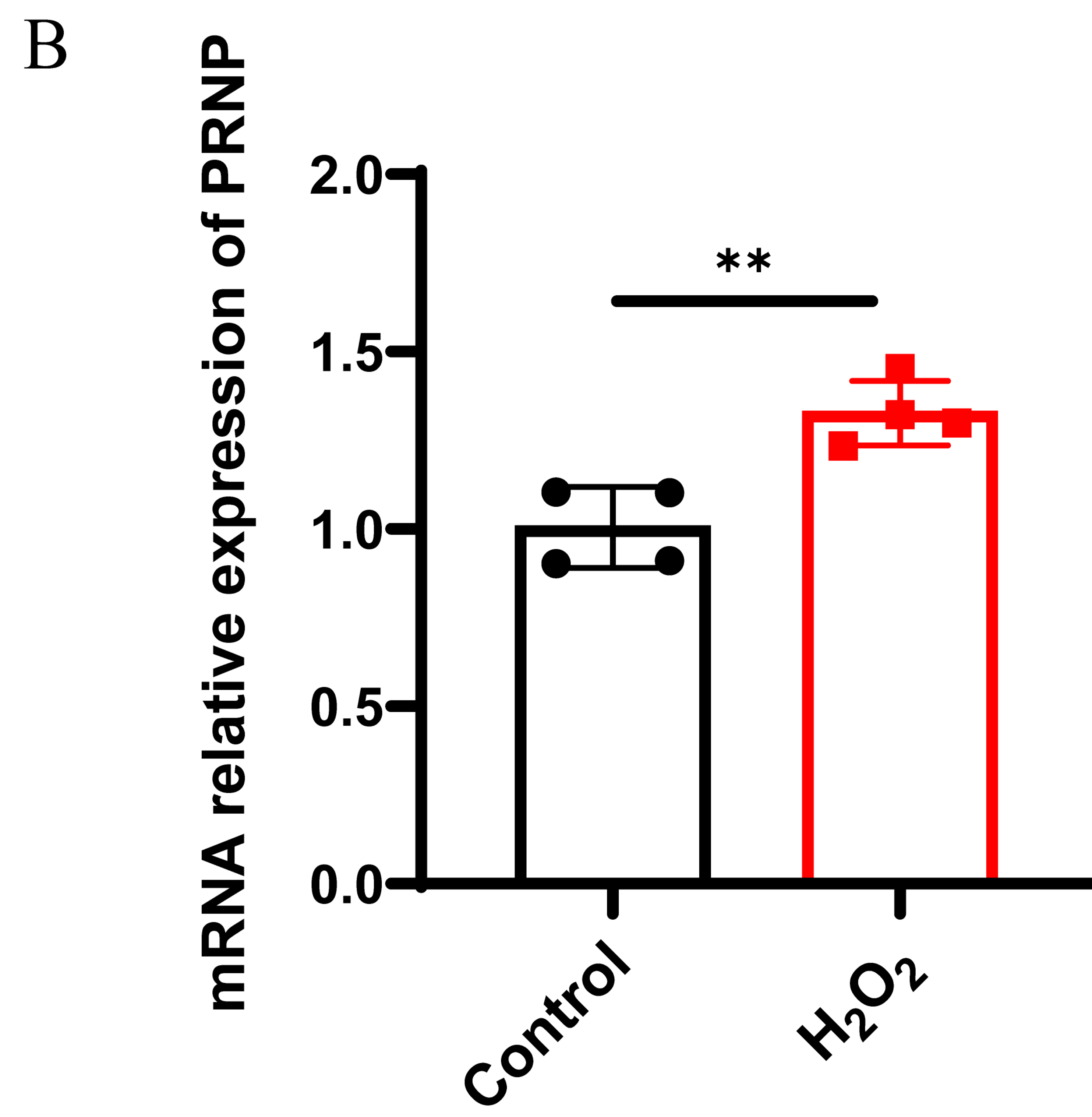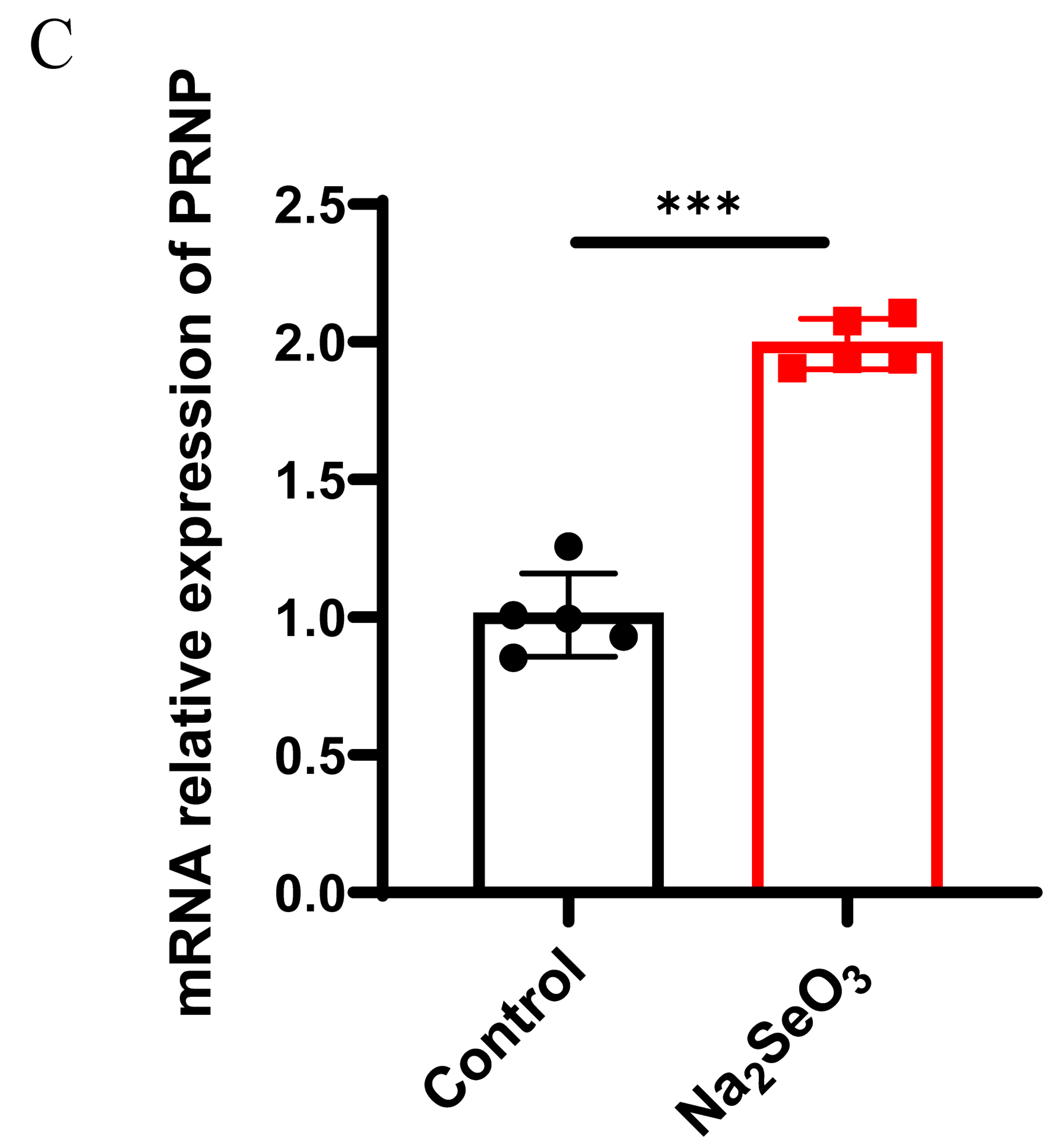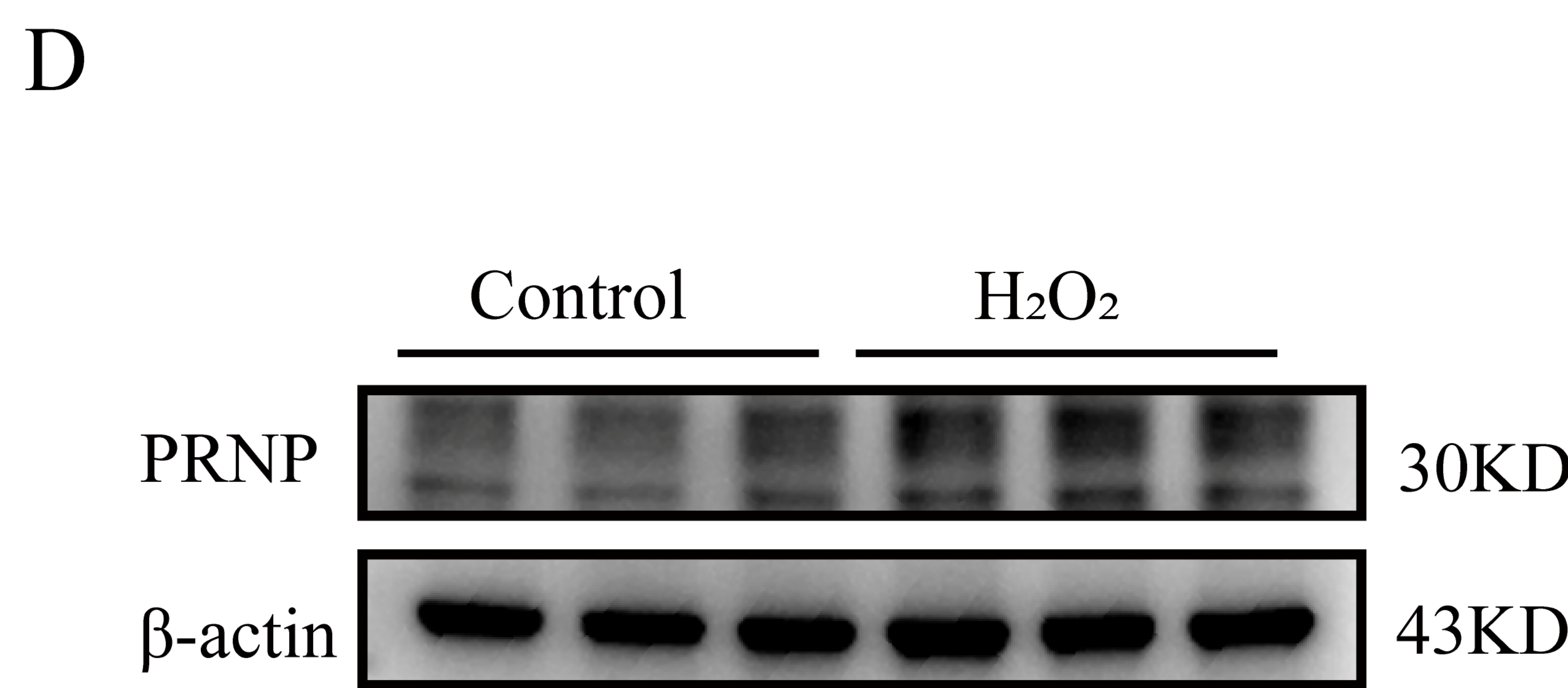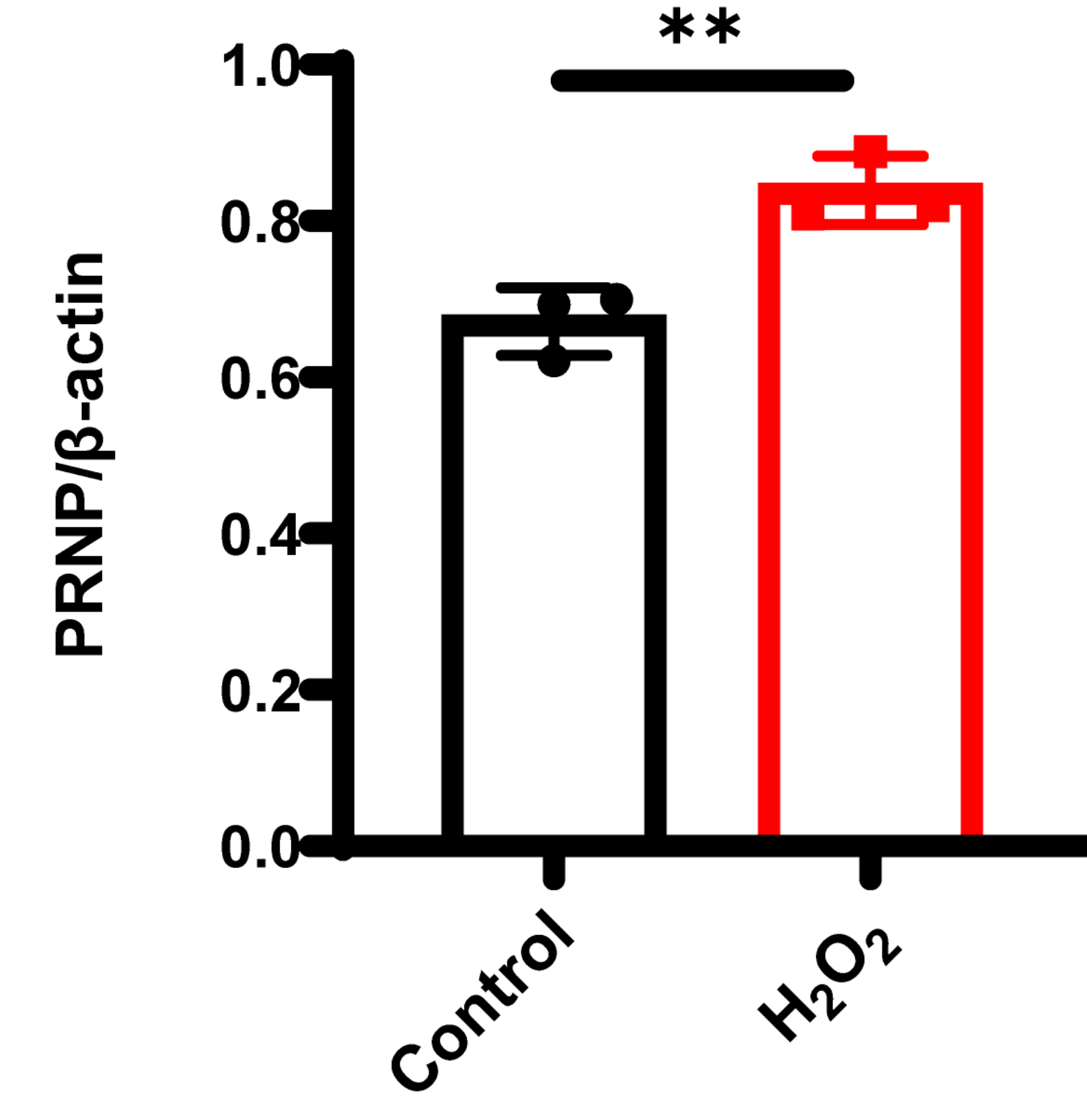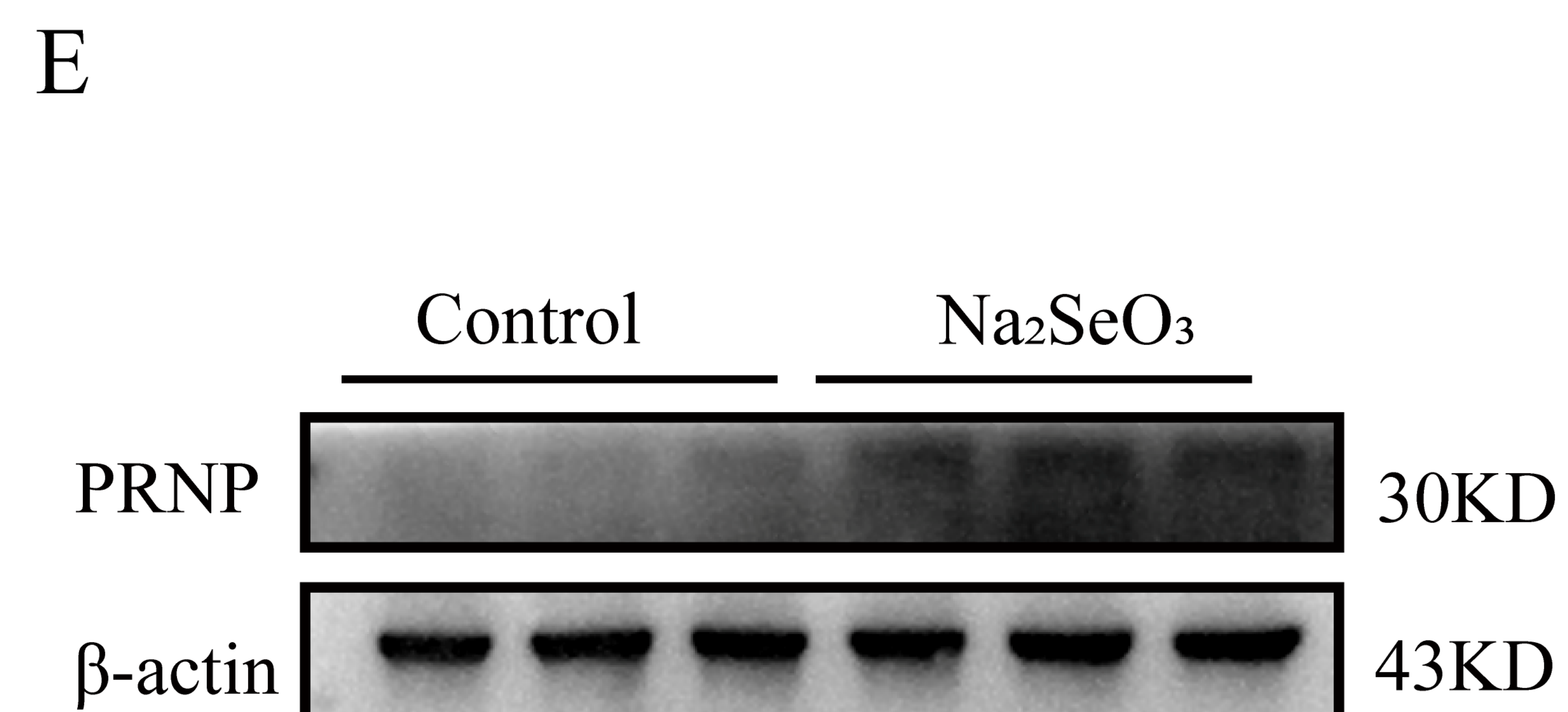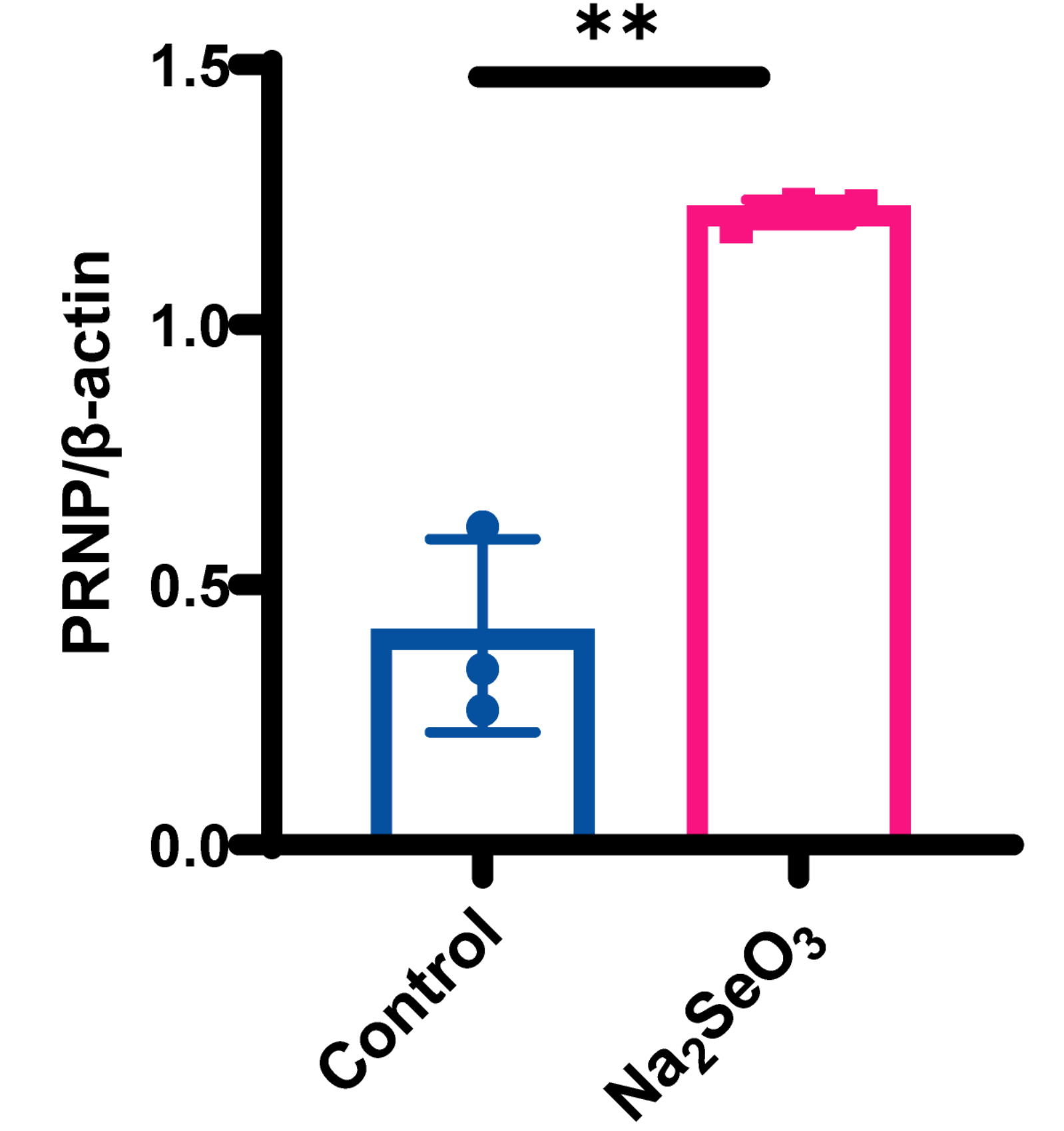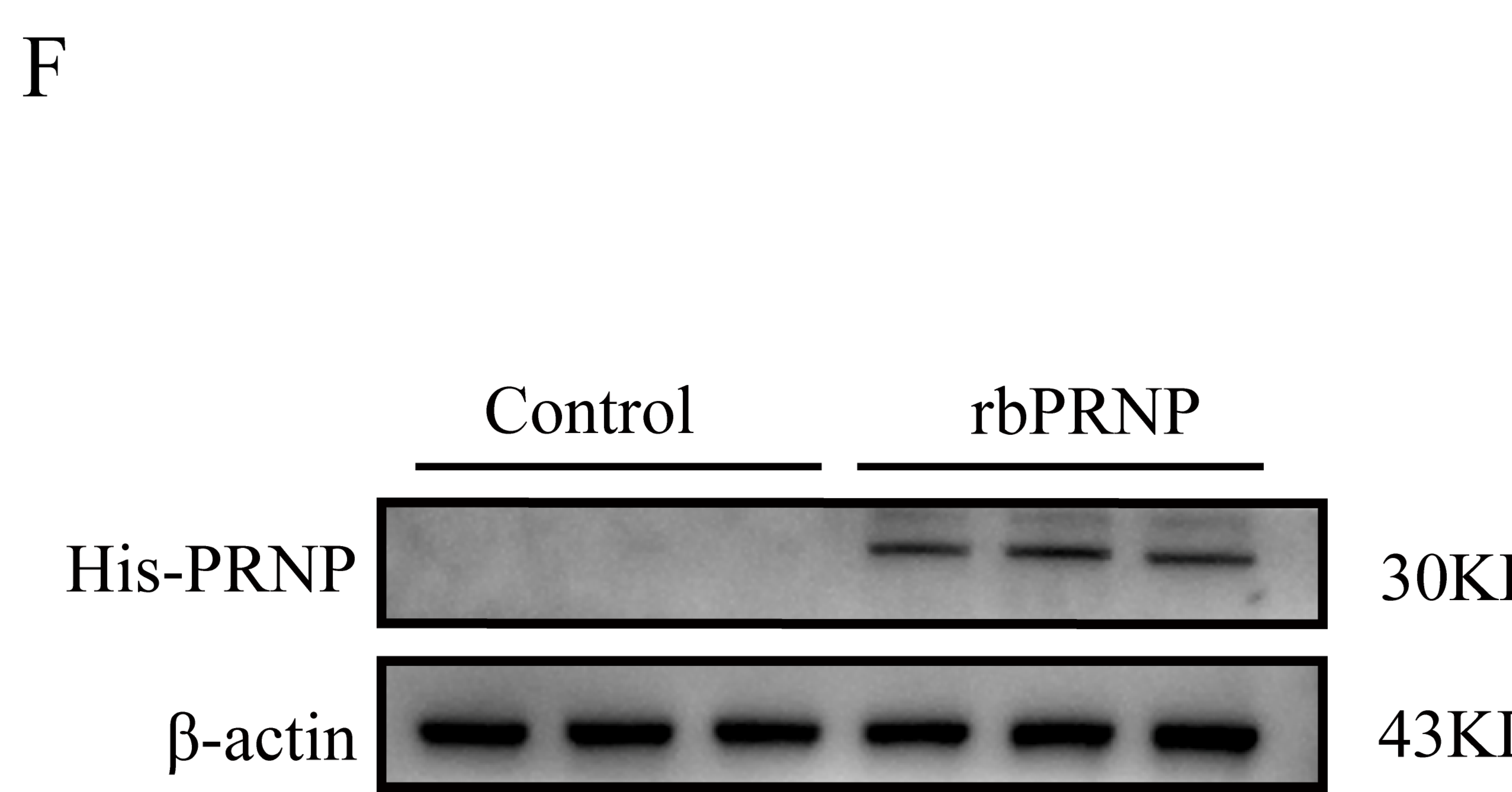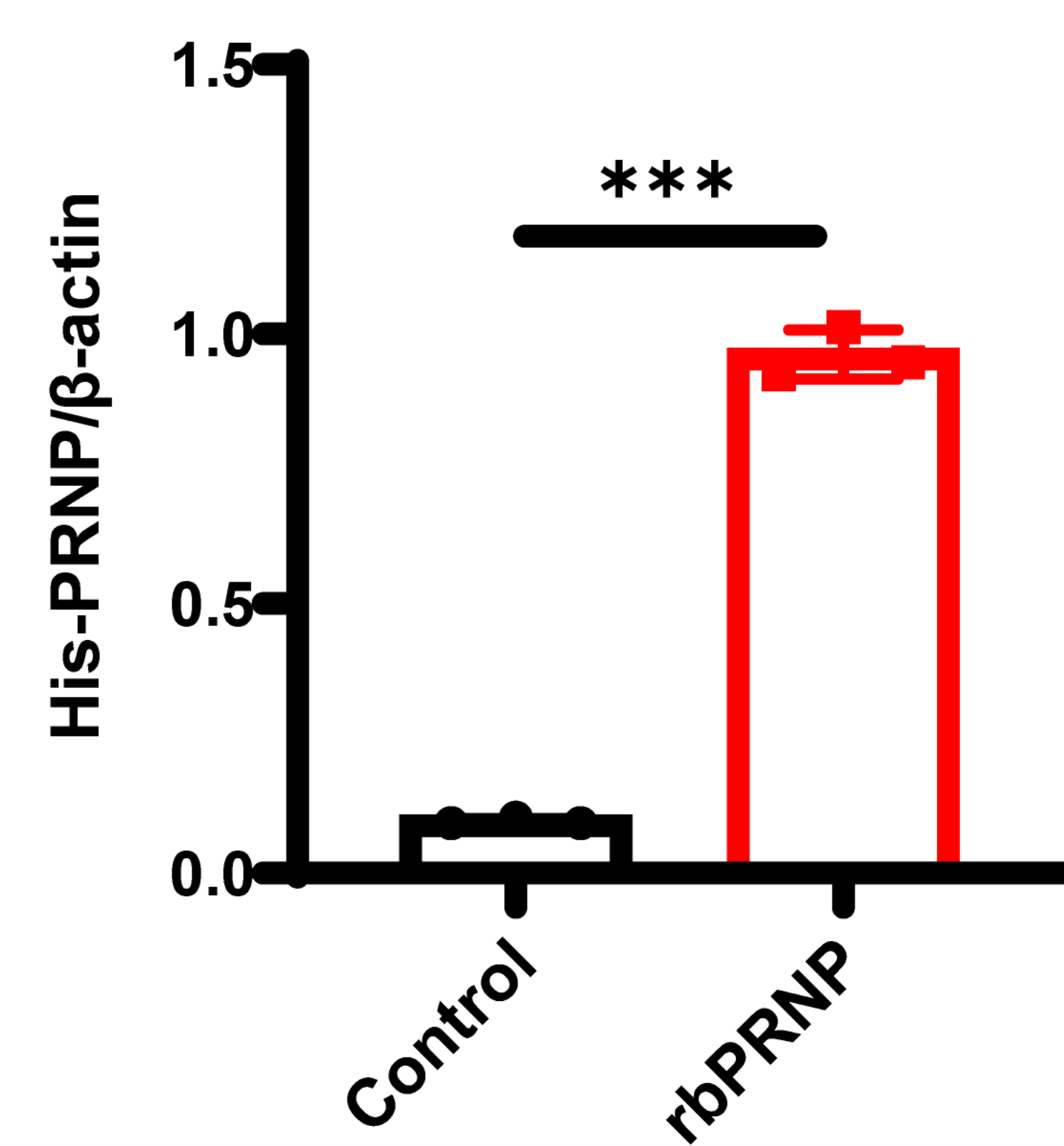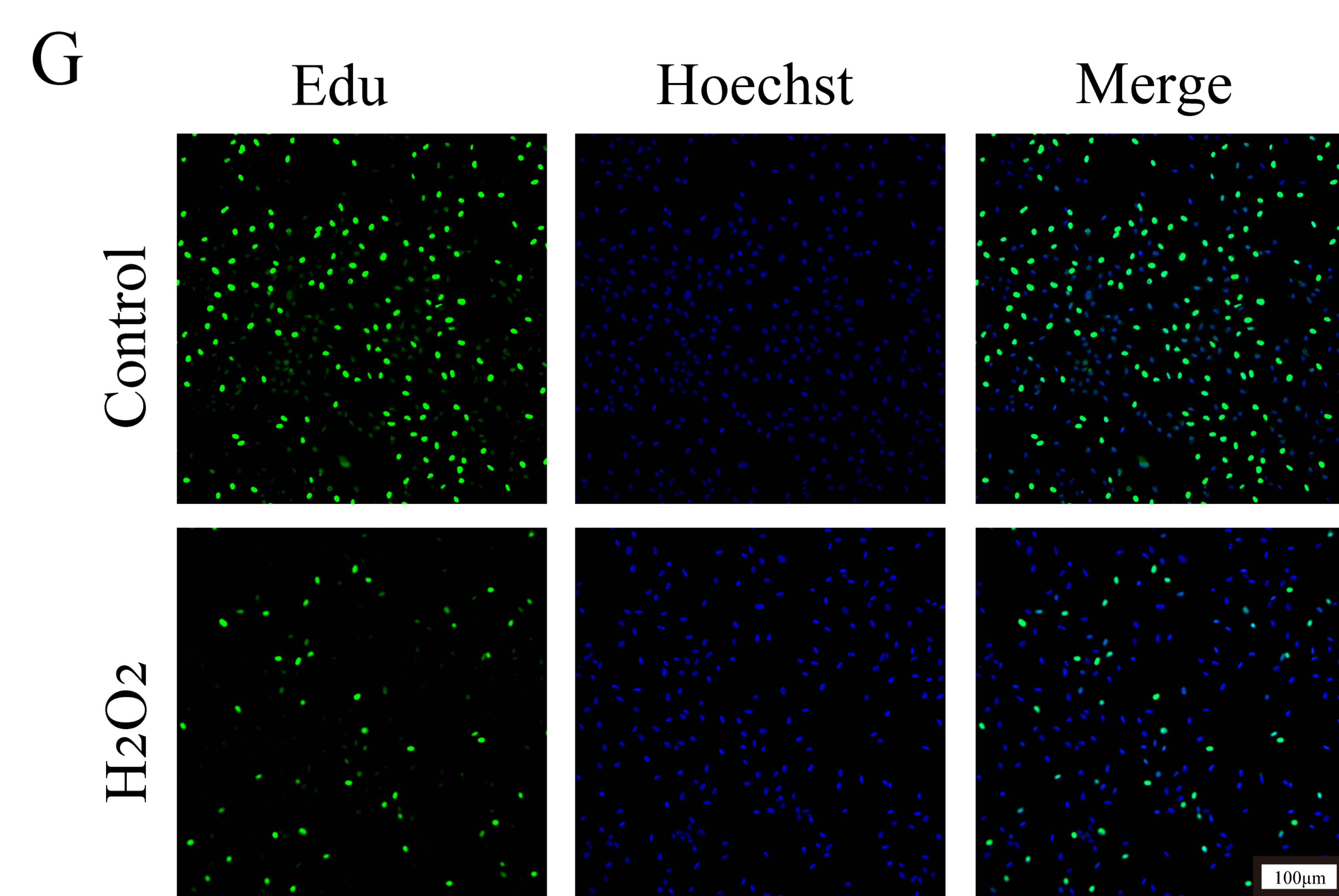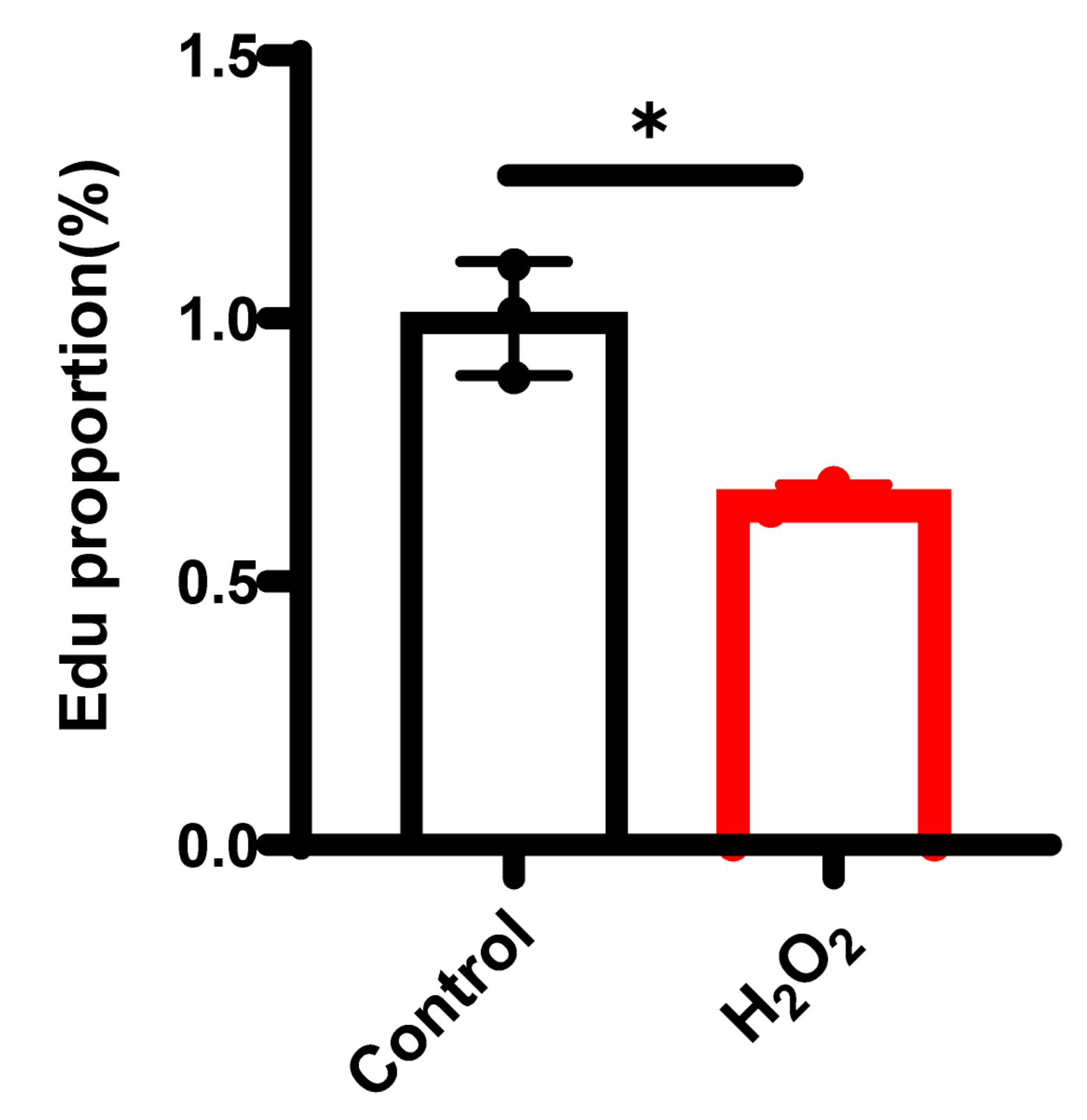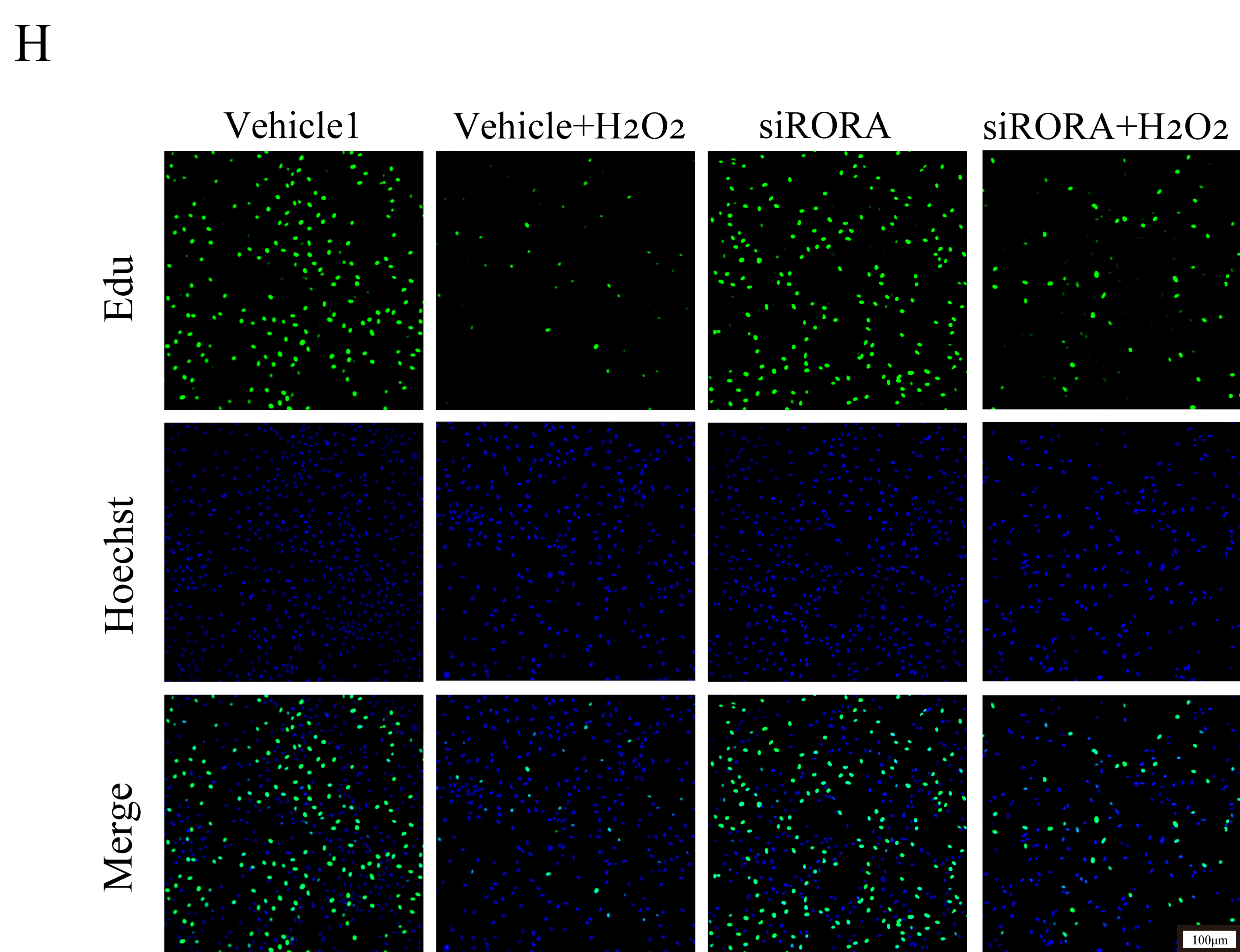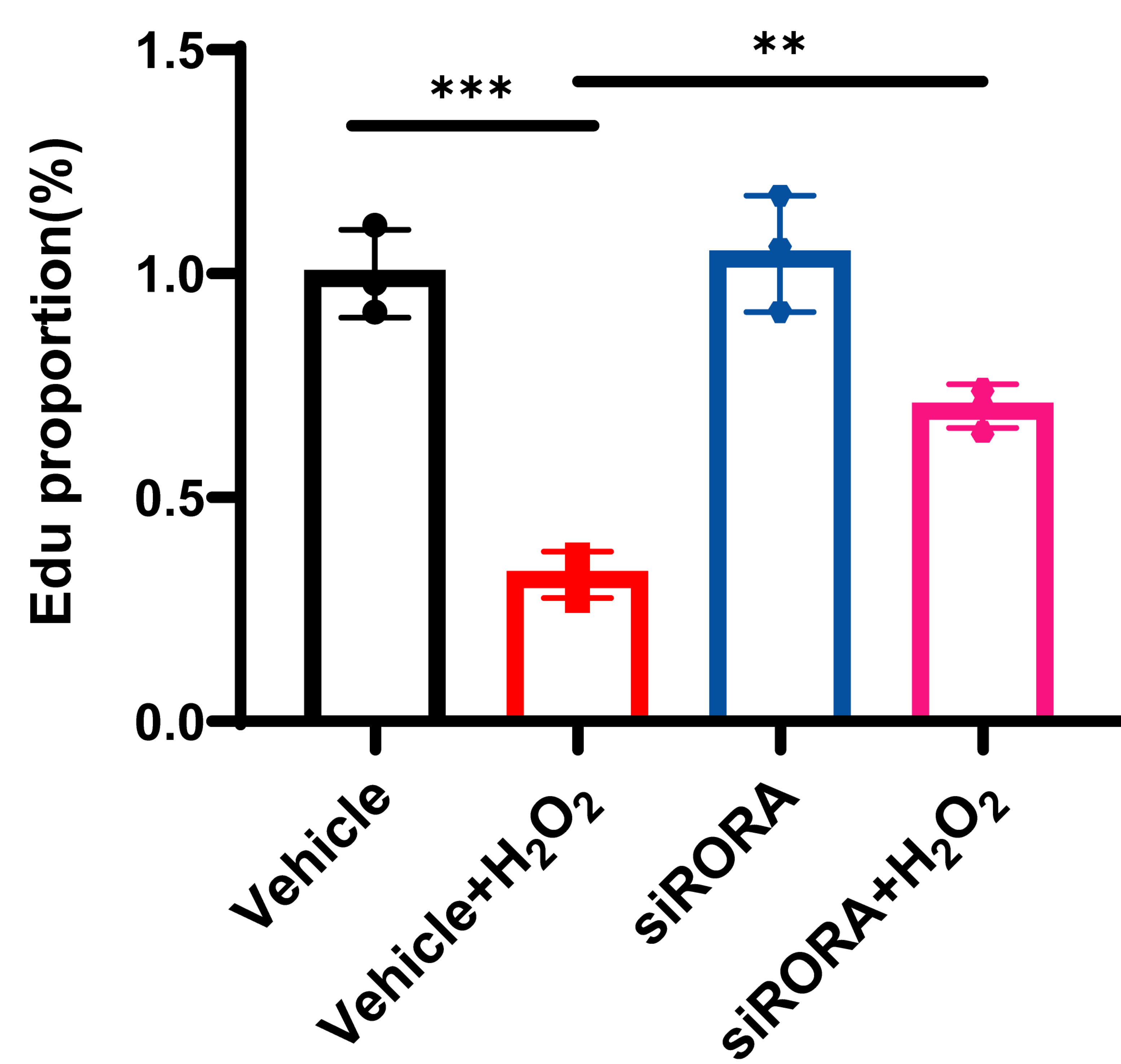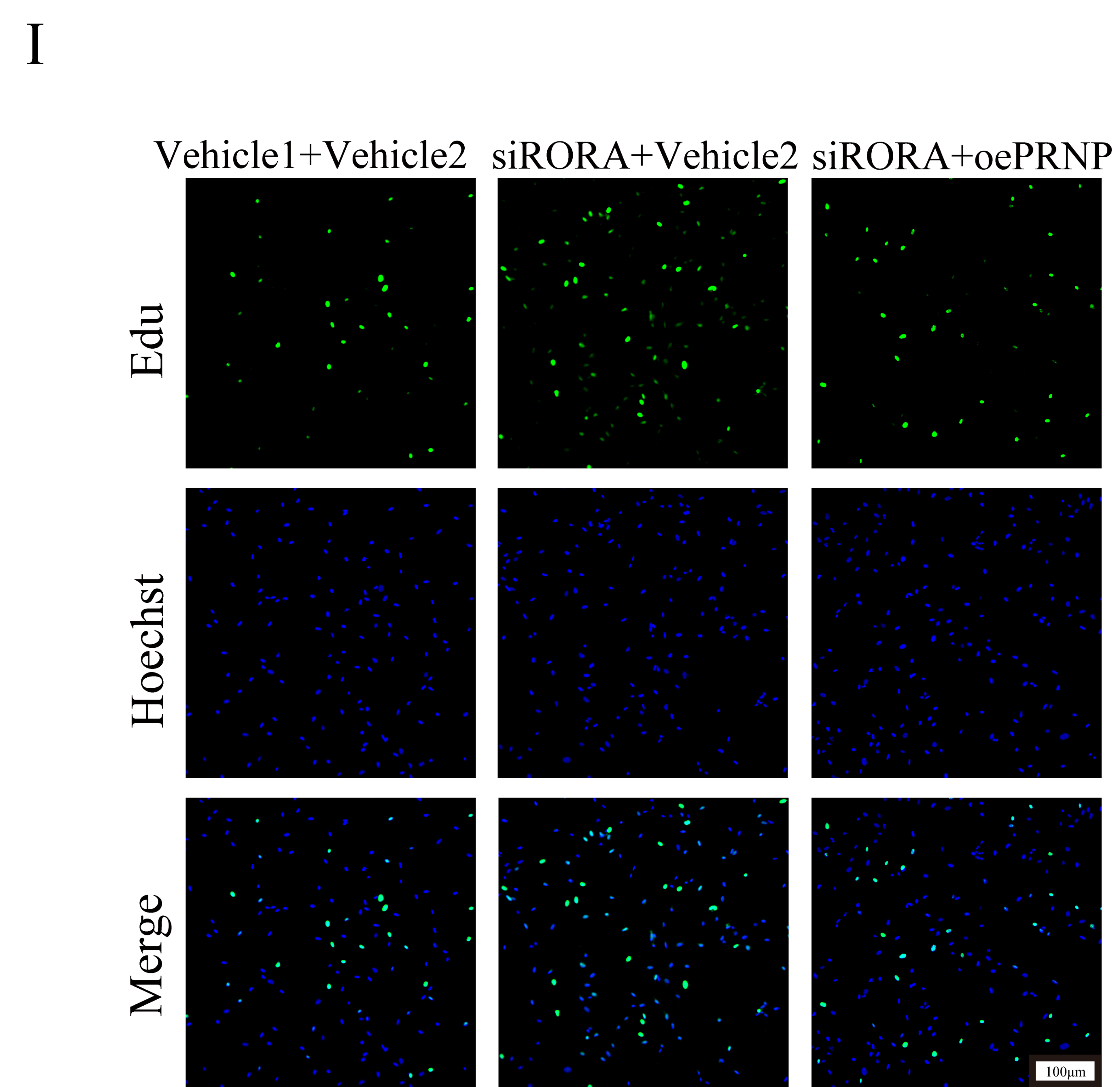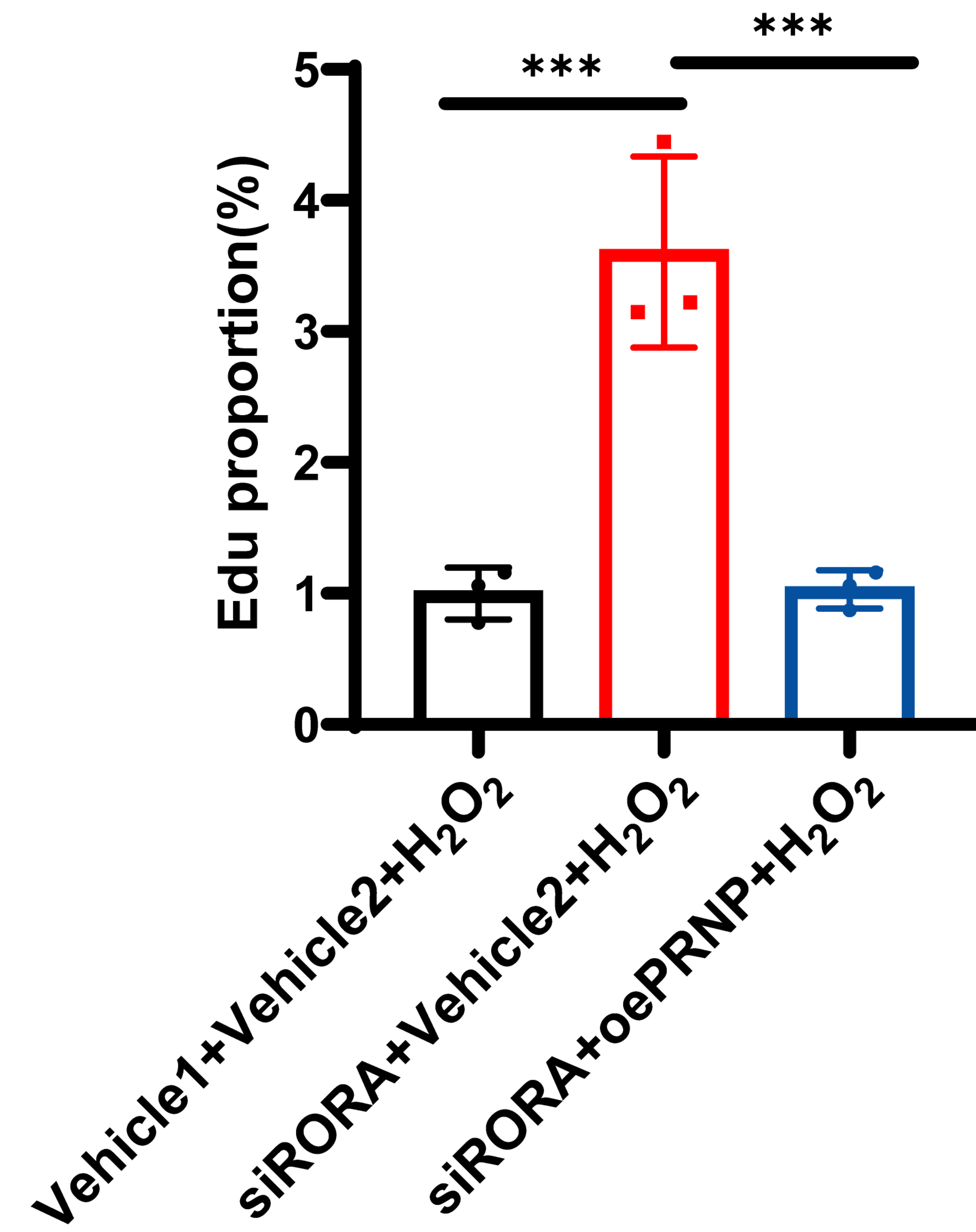

Supplement: Supplementary file 2 — Figure S2: (A) Relative mRNA expression of PRNP in anterior lens capsule tissues from normal controls (n = 5) and ARC patients (n = 8), detected by RT‐qPCR. ***, p < 0.001; unpaired Student's t‐test. (B) Relative mRNA expression of PRNP in Control and H2O2 groups (n = 4). **, p < 0.01; unpaired Student's t‐test. (C) Relative mRNA expression of PRNP in Control and Na2SeO3 groups (n = 5). ***, p < 0.001; unpaired Student's t‐test. (D, E) Relative protein expression of PRNP in each group (n = 3). **, p < 0.01; unpaired Student's t‐test. (F) Relative protein expression of His‐PRNP in rats with or without rbPRNP (n = 3). ***, p < 0.001; unpaired Student's t‐test. (G, H, I) The Edu assays were used to analyze the proliferation of cells in each group (n = 3). Scale bar, 100 μm. [file ACEL-25-e70547-s003.pdf]
