# Supplementary material for: RORA Targeting PRNP Modulates Age‐Related Cataract via Activation Oxidative Injury‐Induced Cellular Senescence and Apoptosis of Lens Epithelial Cells
Source: Aging Cell. 2026 May 20;25(6):e70547. doi: 10.1111/acel.70547 (PMC13239784; doi:10.1111/acel.70547)
Supplement: Supplementary file 3 — Table S1: RT‐qPCR primers used in this study. [file ACEL-25-e70547-s002.docx]

**Table S1. RT-qPCR primers used in this study.**

| **Genes** | **Sequences (5’-3’)** |
| --- | --- |
| β-actin (Human) | Forward: CCTGGACTTCGAGCAAGAGATG |
|  | Reverse: AGGAAGGAAGGCTGGAAGAGTG |
| Bcl-2 (Human) | Forward: TACGAGTGGGATGCGGGAGATG |
|  | Reverse: CCGGGCTGGGAGGAGAAGATG |
| Bax (Human) | Forward: TCAGGATGCGTCCACCAAGAAG |
|  | Reverse: TGTGTCCACGGCGGCAATC |
| RORC (Human) | Forward: AGCGGCAACAGCAGCAACAG |
|  | Reverse: CAGGCAGGTCAGGCGAGGAG |
| RORB (Human) | Forward: AGCAGAGTGGGGAGGCAGAAG |
|  | Reverse: GCCGCTGGTCTCGTTGTTCAG |
| RORA (Human) | Forward: CGGAACTTCACGACGACCTCAG |
|  | Reverse: GGAAGGCTGTATGTCCAGGTAGAAG |
| FLI1 (Human) | Forward: AGAACATGGATGGCAAGGAACTG |
|  | Reverse: CTGAGGTAACTGAGGTGTGACAAC |
| PRNP (Human) | Forward: GCGTGGTTGAGCAGATGTGTATC |
|  | Reverse: GATCACAGGTGGAGAGGAGAAGAG |
| JUNB (Human) | Forward: TTCTGGTCAGGGCTCGGACAC |
|  | Reverse: TGATCACGCCGTTGCTGTTGG |
| CCNE1 (Human) | Forward: AGCCCGAGCAAAGAAAGCCATG |
|  | Reverse: GCTCTGCTTCTTACCGCTCTGTG |
| MYDGF (Human) | Forward: GGACCAGCGAAGACCACCAG |
|  | Reverse: CCTCTGCCTTGAACTGTGTGAAG |
| RELB (Human) | Forward: CAGTGTGTGAGGAAGAAGGAGATTG |
|  | Reverse: CAGGGACCCAGCGTTGTAGG |
| PRNP (Rat) | Forward: ATCACCATCAAGCAGCATACAGTC  Reverse: CCACACGCTCCATCATCTTCAC |
| RORC (Rat) | Reverse: CAGGGACCCAGCGTTGTAGG |
|  | Reverse: TGGCTGCGGCGGAAGAAG |
| RORB (Rat) | Forward: CGTGCCTTCAACCCATTAAACAAC |
|  | Reverse: TGCTTCATTCACTAGGTCATCAGAAC |
| RORA (Rat) | Forward: AGCAGCAGCAGCGAGACC |
|  | Reverse: GCAGTTCCGTCAGCCCATTG |
| Bcl-2 (Rat) | Forward: AGAGGGGCTACGAGTGGGATAC |
|  | Reverse: GGTTGCTCTCAGGCTGGAAGG |
| Bax (Rat) | Forward: GGAGACACCTGAGCTGACCTTG |
|  | Reverse: TCGCCAATTCGCCTGAGACAC |
| β-actin (Rat) | Forward: CTATCGGCAATGAGCGGTTCC |
|  | Reverse: GCACTGTGTTGGCATAGAGGTC |
