# Supplementary material for: RORA Targeting PRNP Modulates Age‐Related Cataract via Activation Oxidative Injury‐Induced Cellular Senescence and Apoptosis of Lens Epithelial Cells
Source: Aging Cell. 2026 May 20;25(6):e70547. doi: 10.1111/acel.70547 (PMC13239784; doi:10.1111/acel.70547)
Supplement: Supplementary file 1 — Figure S1: RORA activation exacerbates H2O2‐induced cellular senescence, apoptosis and oxidative damage in vitro. (A) Viability of LECs treated with increasing concentrations of the RORA activator SR1078, measured by CCK‐8 assay (n = 3). *, p < 0.05; ***, p < 0.001; ns, p > 0.05; unpaired Student's t‐test. (B, C) RORA activation efficiency assessed by RT‐qPCR (mRNA level) and Western blot (protein level) (n = 3). *, p < 0.05; **, p < 0.01; ***, p < 0.001; one‐way ANOVA. (D) Fluorescence images and relative fluorescence intensity analysis of ROS assay results (n = 3). Scale bar, 100 μm. ***, p < 0.001; one‐way ANOVA. (E) Mitochondrial membrane potential in each of the three groups determined by the JC‐1 probe (n = 3). Scale bar, 50 μm. (F, G) MDA content and SOD activity in the three groups: Control, H2O2 and H2O2 + SR1078 groups (n = 3). *, p < 0.05; **, p < 0.01; ***, p < 0.001; one‐way ANOVA. (H) Relative protein expression levels of apoptosis‐related factors (Bcl‐2, Bax, Cleaved‐C3) and senescence factors (P21, P53) in the three groups (n = 3). *, p < 0.05; **, p < 0.01; ***, p < 0.001; one‐way ANOVA. [file ACEL-25-e70547-s001.pdf]

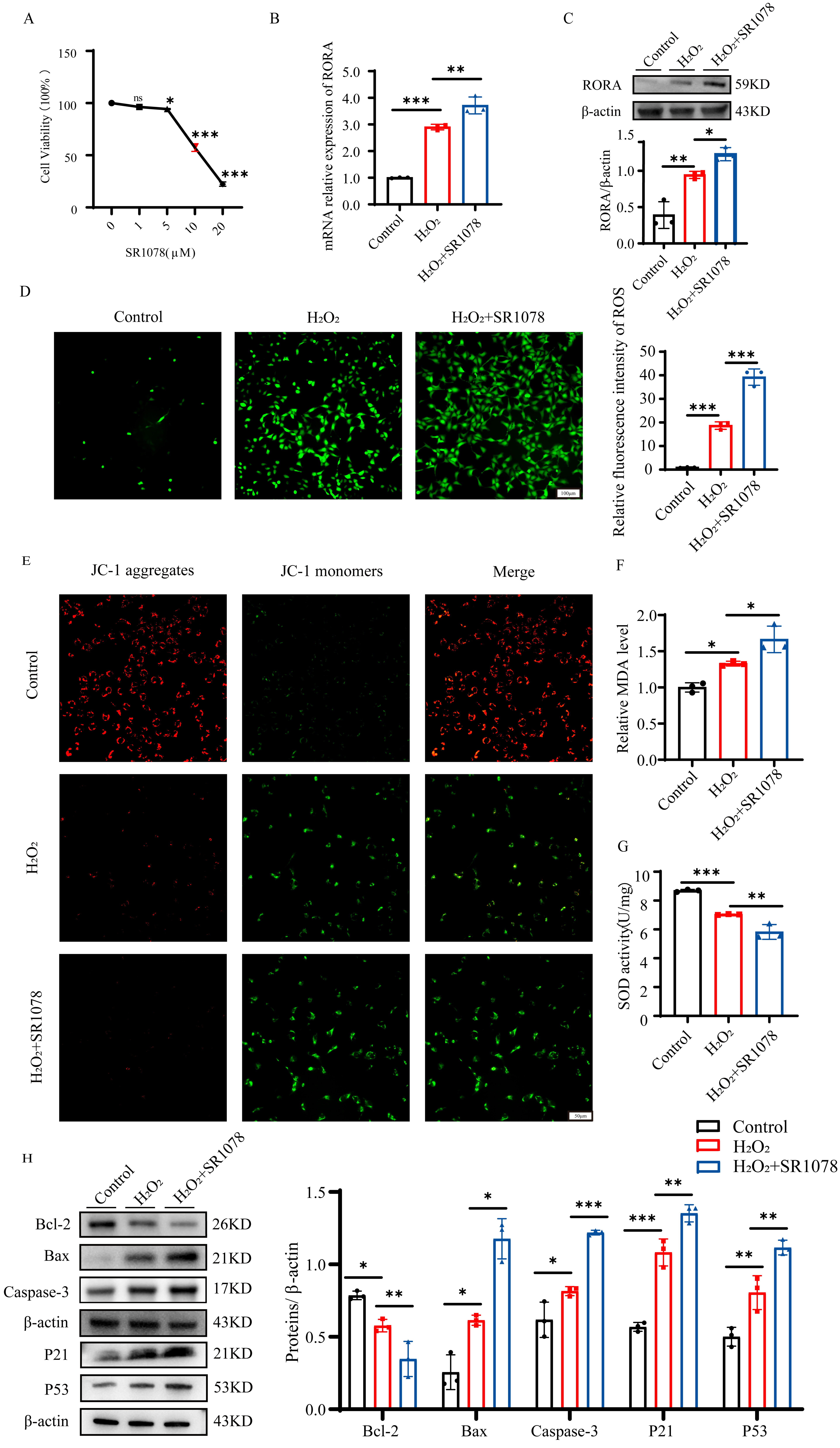

A
Cell Viability (100%)
SR1078(μM)
ns
B
mRNA relative expression of RORA
Control
H_2O_2
H_2O_2+SR1078
C
RORA
β-actin
59KD
43KD
RORA/β-actin
D
Relative fluorescence intensity of ROS
100μm
E
JC-1 aggregates
JC-1 monomers
Merge
50μm
F
Relative MDA level
G
SOD activity(U/mg)
H
Bcl-2
Bax
Caspase-3
P21
P53
26KD
21KD
17KD
53KD
Proteins/ β-actin
